# Apple miRNAs and tasiRNAs with novel regulatory networks

**DOI:** 10.1186/gb-2012-13-6-r47

**Published:** 2012-06-15

**Authors:** Rui Xia, Hong Zhu, Yong-qiang An, Eric P Beers, Zongrang Liu

**Affiliations:** 1 Alson H Smith Agricultural Research and Extension Center, Department of Horticulture, Virginia Polytechnic Institute and State University, Winchester, VA 22602, USA; 2 Department of Horticulture, Virginia Polytechnic Institute and State University, Blacksburg, VA 24061, USA; 3 Appalachian Fruit Research Station, Agricultural Research Service, United States Department of Agriculture, Kearneysville, WV 25430, USA; 4 Plant Genetics Research Unit, Agricultural Research Service, United States Department of Agriculture, Donald Danforth Plant Science Center, St Louis, MO 63132, USA

## Abstract

**Background:**

MicroRNAs (miRNAs) and their regulatory functions have been extensively characterized in model species but whether apple has evolved similar or unique regulatory features remains unknown.

**Results:**

We performed deep small RNA-seq and identified 23 conserved, 10 less-conserved and 42 apple-specific miRNAs or families with distinct expression patterns. The identified miRNAs target 118 genes representing a wide range of enzymatic and regulatory activities. Apple also conserves two *TAS *gene families with similar but unique *trans*-acting small interfering RNA (tasiRNA) biogenesis profiles and target specificities. Importantly, we found that miR159, miR828 and miR858 can collectively target up to 81 *MYB *genes potentially involved in diverse aspects of plant growth and development. These miRNA target sites are differentially conserved among *MYB*s, which is largely influenced by the location and conservation of the encoded amino acid residues in MYB factors. Finally, we found that 10 of the 19 miR828-targeted *MYB*s undergo small interfering RNA (siRNA) biogenesis at the 3' cleaved, highly divergent transcript regions, generating over 100 sequence-distinct siRNAs that potentially target over 70 diverse genes as confirmed by degradome analysis.

**Conclusions:**

Our work identified and characterized apple miRNAs, their expression patterns, targets and regulatory functions. We also discovered that three miRNAs and the ensuing siRNAs exploit both conserved and divergent sequence features of *MYB *genes to initiate distinct regulatory networks targeting a multitude of genes inside and outside the *MYB *family.

## Background

The discovery of RNA interference in the late 1990s [1] prompted a revolution in RNA biology, and the unveiling of small RNA (sRNA)-mediated gene regulatory pathways has profoundly shaped our understanding of the complexity of gene regulation. In eukaryotes, sRNAs have been found to control cellular metabolism, growth and differentiation, to maintain genome integrity, and to combat viruses and mobile genetic elements [2]. These regulatory sRNAs have been classified into at least six groups, including microRNAs (miRNAs), heterochromatic small interfering RNAs (hc-siRNAs), *trans*-acting small interfering RNAs (tasiRNAs), natural antisense small interfering RNAs (nat-siRNAs), repeat-associated small interfering RNAs (ra-siRNAs), and in metazoans, the piwi-interacting RNAs (piRNAs) [3-7].

miRNAs are derived from single-stranded RNA precursors that are transcribed by RNA polymerase II to generate self-complementary fold-back structures (stem-loop or hairpin) processed subsequently by DICER-like 1 (DCL1) in association with other protein factors [6,8]. Distinct from miRNA biogenesis, small interfering RNAs (siRNAs) are generated from long double-stranded RNAs that are converted from single-stranded RNAs by plant RNA-dependent RNA polymerases (RDRs), which usually give rise to transcript-wide, distinct siRNA species from both strands dependent on the choice of DCL proteins involved [8]. Biogenesis of predominant small 21-nucleotide siRNAs requires RDR6 and DCL4 while that of predominant 24-nucleotide siRNAs requires RDR2 and DCL3 [9,10]. miRNAs and siRNAs are incorporated into different RNA-induced silencing complexes (RISCs) [11], where one of the Argonaute (AGO) factors catalyzes sequence-specific endonucleotic cleavage of targeted gene transcripts [12,13] in the cases of miRNAs and 21-nucleotide siRNAs [11], or translational repression occasionally for some miRNAs [14], or induction of DNA methylation in the case of 24-nucleotide siRNAs [15,16]. The biogenesis of tasiRNAs exploits both miRNA and 21-nucleotide siRNA biogenesis pathways, and requires all the factors necessary for miRNA and 21-nucleotide siRNA production, including DCL1, RDR6 and DCL4 as well as other protein factors [5,17]. In *Arabidopsis*, the transcript from a *trans*-acting siRNA (*TAS*) gene is first cleaved by one of three specialized miRNAs (miR173, miR390 and miR828), and then either the 3'-cleaved (in the case of miRNA828, miR173) or the 5'-cleaved transcript fragments (in the case of miR390) are converted into double-stranded RNAs by RDR6 and subsequently diced into phased 21-nucleotide siRNAs by DCL4 to generate multiple but distinct tasiRNA species, some of which are found to further guide sequence-specific cleavage of their targeted gene transcripts through the RISC [5,17-20]. To date, only four *TAS *gene families have been identified in *Arabidopsis *and their biogenesis has been extensively characterized [21].

In plants, miRNAs are the second most abundant sRNAs [22], acting as powerful endogenous regulators. For example, many distinct miRNAs target transcripts encoding an array of transcription factors that control plant development and phase transition in *Arabidopsis*, maize and woody species [23-25], while others are involved in stress response and disease resistance [26-28]. In humans, it is estimated that at least 30% of genes are regulated by miRNAs [29], further underscoring their fundamental importance. Whether a similar proportion of plant genes are subjected to miRNA-mediated regulation is unknown; however, a large number of miRNAs have been identified, characterized and reported in diverse plant species, including *Arabidopsis *[30,31], rice [32], maize [33], poplar [34,35], grape [36], soybean [37], orange [38] and peanut [39]. The latest release of published miRNAs (miRBase 17) contains over 15,000 miRNA gene loci in over 140 species, and over 17,000 distinct mature miRNA sequences [40]. Like many gene regulatory systems, miRNAs show both conservation and diversity among plant lineages. Many miRNAs are conserved in angiosperms or even embryophyta [41], while a significant number of miRNAs or miRNA families show species-specificity, reflecting their fast evolving, functionally diverging natures [11,41-43].

Apple (*Malus × domestica*) is a major temperate fruit crop worldwide. Its fruit is a widely consumed and rich source of phytochemicals, which may play a key role in reducing chronic disease risk in humans [44]. As a perennial species, apple undergoes many distinct developmental programs and inducible responses during its life cycle that cannot be easily replicated or investigated in annual model species such as *Arabidopsis*. For example, apple requires a long period of juvenility (5 to 7 years) before flowering [45] and its reproductive cycle lasts for nearly a year, as fruit forms from flower buds initiated during the previous summer. Its fruit development, which spans the spring, summer and fall seasons, comprises fruit enlargement, color changes, texture improvement and ripening, all of which are directly relevant to crop productivity and quality [45]. That apple trees produce fruit over a period spanning several decades is another important consideration for investigation of plant longevity. Thus, apple trees represent an important model for investigating the fundamental biology of a wide range of specialized strategies and programs to adapt or respond to seasonal and perhaps climatic changes as well as biotic and abiotic stress challenges while implementing multiple coordinated developmental events necessary for perennial fruit production. In addition to its importance as a new genomic model for tree fruit and Rosaceae studies, the discovery of genetic mechanisms that regulate fruit development and quality or stress responses and disease resistance could enhance the molecular breeding of apple for horticulturally important traits.

Although several groups have recently reported bioinformatic prediction and identification of a few miRNA families for apple [46,47], nearly all those reported are conserved miRNAs. Whether apple has evolved specific miRNAs and unique regulatory networks, which genes they target, if any, and what biological processes they regulate remain largely unknown. In this study, as part of a long-term goal to elucidate the role of miRNAs in apple, we exploited deep sequencing and computational and molecular analyses to comprehensively identify the conserved and apple-specific miRNAs and their targets, and characterized their expression in various tissues. We also delineated novel miRNA- and tasiRNA-mediated regulatory networks that modulate a large number of genes inside and outside the *MYB *family, which has not been reported in other species.

## Results

### Identification of conserved and less-conserved miRNAs in apple

**Figure 1:**
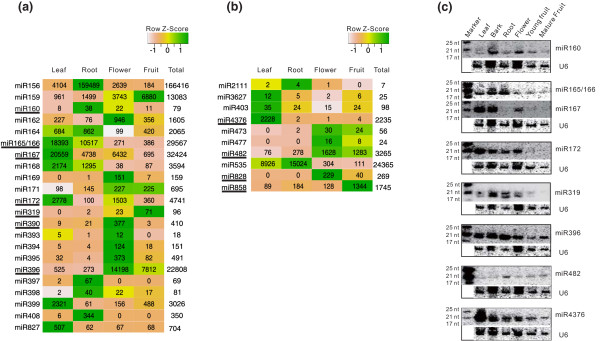
**Analysis of the identified conserved and less-conserved miRNA expression in apple tissues. (a, b) **Heat map and read abundance of the conserved (a) and less-conserved (b) miRNAs in four tissues. The read abundance was normalized and expressed as reads per million (RPM) of genome-matched reads in each tissue. Only reads having two or fewer mismatches to miRNA sequences were counted for this analysis. Read abundance is also denoted by color, as illustrated at the top-right corner of each panel. miRNAs selected for validation by RNA gel blot analysis are underlined. **(c) **RNA gel blot analysis of selected miRNAs in different tissues. Sizes of small RNA markers are indicated at left in nucleotides (nt). Blotting results from probing and reprobing the same filter were grouped together. The apple *U6 *gene serves as a loading control.

A total of 59 million reliable reads were obtained from deep sequencing of leaf-, root-, flower- and fruit-derived sRNA libraries, and most of these reads (about 78% for redundant reads and 65% for unique reads) have at least one perfect match to the apple genome (Table S1 in Additional file 1). sRNAs from each library or tissue shared a more or less similar distribution pattern (Figures S1a-f in Additional file 2), with 24-nucleotide sRNAs (over 40%) being the most abundant followed by 21-nucleotide sRNAs, consistent with earlier findings in *Arabidopsis *[30], tomato [48], and soybean [37]. Those reads (46 million) that perfectly mapped to the apple genome were subjected to further analyses for miRNA identification. As a result, we identified 23 miRNAs or families (Figure 1a) that are conserved in both angiosperms and coniferophyta lineages [41]; we refer to these as conserved miRNAs in this study. These miRNAs bore a canonical stem-loop structure in their precursors (Table S2 in Additional file 1). Expression levels of the conserved miRNAs, as reflected by normalized reads (reads per million genome-matched reads (RPM)), showed a great variation among families in all four tissues. The highest read abundance (166,000 RPM) was detected for miR156 and was 5 to 16 times more than other relatively abundant miRNA families, including miR165/166, miR167, miR396, and miR159, whose total abundance ranged from 10,000 to 30,000 RPM (Figure 1a; Table S3 in Additional file 1). Although lower expression (between 1,500 and 4,000 RPM) was observed for the miR162, miR164, miR168, miR172 and miR399 families, their overall expression level was 3 to 20 times greater than any of the remaining 12 conserved miRNA families (Figure 1a; Table S3 in Additional file 1). Almost all miRNAs showed, to various degrees, differential expression among the tissues analyzed, with the greatest variation observed for miR156, which was expressed at an abundance of more than 150,000 RPM in root but only 184 RPM in fruit (Figure 1a). Most of the conserved miRNA families comprised multiple species of different mature sequences (≤2 mismatches) and distinct length predominance (Tables S3 and S4 in Additional file 1).

We also identified ten miRNAs or families that have a canonical stem-loop structure (Figure 1b; Table S2 in Additional file 1) and were reported in some plant species or families but are not widely conserved in both angiosperm and coniferophyta lineages [41]. They are referred to as less-conserved miRNAs in this study. Compared to the conserved miRNAs, most of the less-conserved miRNAs exhibited relatively lower expression, with the most notable exception being miR535, which was expressed at an abundance of more than 20,000 RPM (Figure 1b). However, these less-conserved miRNAs, like the conserved miRNAs, were differentially regulated among the tissues examined. For example, leaf- and root-biased expression was observed for miR535, while flower-biased expression was apparent for miR828 (Figure 1b). Surprisingly, miR4376 exhibited virtually exclusive expression in leaf tissues where 2,228 RPM were detected in comparison with less than 4 RPM in other tissues.

**Figure 2:**
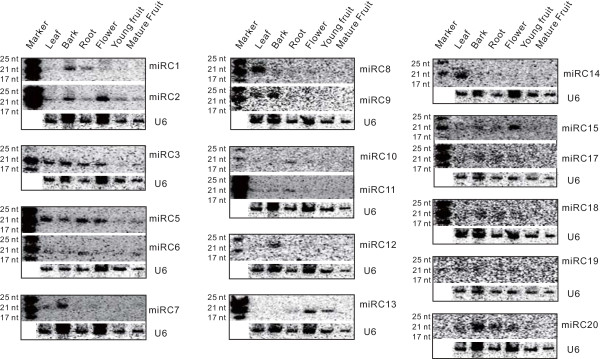
**Confirmation of apple-specific miRNA expression by RNA gel blot analysis**. RNA gel blot analysis was performed as described in Materials and methods with exposure time varying from a few hours to two to three days for some low-abundance miRNAs (miR18, miR19, miR20). The blotting results from the same filter were grouped together. Sizes of small RNA markers are indicated at left in nucleotides (nt). The apple *U6 *gene serves as a loading control.

**Figure 3:**
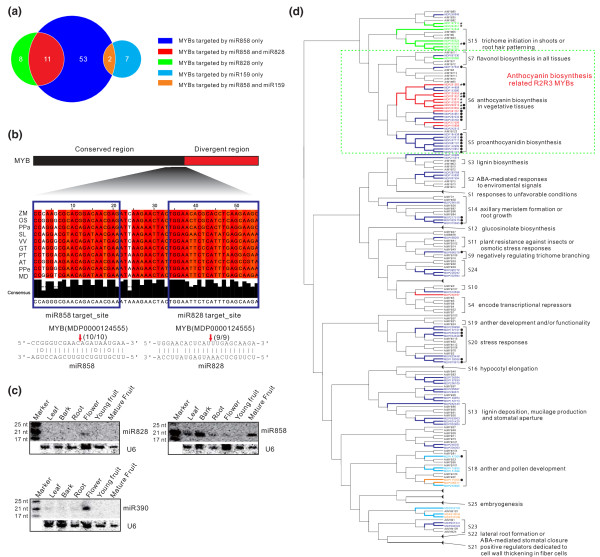
**Complex *MYB *regulatory network mediated by miR159, miR828, and miR858**. **(a) **Numbers of *MYB *genes targeted by apple miR159, miR828 and miR858. **(b) **Conservation of the region co-targeted by miR828 and miR858 among diverse plant species, including *Zea mays *(ZM; BT088210), *Oryza sativa *(OS; NM_001050499), *Physcomitrella patens *(PPa; XM_001781987), *Solanum lycopersicum*(SL; AK322126), *Vitis vinifera *(VV; XM_002269386), *Gossypium tomentosum *(GT; EU249416), *Populus trichocarpa *(PT; XM_002315854), *Arabidopsis thaliana *(AT; NM_116126), *Prunus persica *(PPe; ppa016135m), and *Malus × domestica *(MD; MDP0000124555). The sequence-conserved region in the *MYB *coding region is shown in black while the sequence-divergent region is shown in red. Pairing of miR828 and miR858 with their target sites in a representative *MYB *transcript is illustrated below. **(c) **RNA gel blot analysis of expression of miR390, miR828 and miR858 in various tissues. **(d) **Phylogenetic analysis and functional relationship between apple and *Arabidopsis *MYB factors. The tree was inferred using the neighbor-joining method and 1,000 bootstraps with MYB amino acid sequences and Clustal X2 software. The subgroup and function annotation were designated as described previously [56,58]. Subclans consisting of only *Arabidopsis *MYBs were collapsed are and denoted with black triangles. The *MYB *genes that are targeted by a specific miRNA or co-targeted by two miRNAs are denoted in a specific color as designated in (a). miR828-targeted *MYB*s capable of producing secondary siRNAs are marked with a star, and *MYB*s whose cleavage was confirmed by degradome analysis are marked with filled circles. ABA, abscisic acid; nt, nucleotide.

To validate miRNA RPM data, we performed RNA gel blot analysis for selected miRNAs representing conserved, less-conserved and apple-specific (discussed below) examples in six different tissues, four of which (leaf, root, flower and fruit) could be compared to sRNA sequencing data (Figures 1c, 2, and 3c). We found that while blotting results for some miRNAs - miR828, miR858, miR390 (Figure 3c) and miR4376 (Figure 1c) - were reflective of the relative abundances of sequenced RNAs from these four tissues, many others displayed varying degrees of divergence between the two analyses. For example, miR172 RPM values and blot signals for leaf and flower were in agreement, while the blot signal for fruit, which should be nearly four-fold higher than for root, based on RPM values, was barely detectable. Additionally, miR396, which showed relative blot signal strength that was high for vegetative and low for reproductive tissues, revealed the opposite pattern through RNA sequencing (Figure 1a). At present we do not know why some RNA sequencing values were corroborated by RNA blots while others were not. However, contradictions between *in vivo *RNA levels and sequencing results for miRNAs have been previously reported for *Arabidopsis *[30] and grapevine [36]. Since the hybridization signal of RNA gel blotting is proportional to gene transcript abundance in general, the bias introduced against or for certain sequences or sequence motifs during either library construction or sequence amplification or deep sequencing may have contributed to the observed deviations.

### Apple-specific miRNAs

Since numerous family- or species-specific miRNAs considered to be of a more recent evolutionary origin [11] have been identified in other species, apple is likely to have evolved unique miRNAs as well. After excluding sRNA reads homologous to known miRNAs (two or fewer mismatches, miRBase 17) and other non-coding sRNAs (Rfam 10), the remaining 20- to 22-nucleotide sRNAs were subjected to rigorous secondary structural analysis of their precursors using RNAfold software [49]. Those precursors with a canonical stem-loop structure were further analyzed through a series of stringent filter strategies to ensure that they met common criteria established by the research community [50,51]. A total of 42 miRNA candidates derived from 75 loci (Tables 1 and S5 in Additional file 1) met the screening criteria, of which 21 had miRNA star (miRNA*) sequences identified from the same libraries, while the other 21 had no miRNA* identified (Table 1). We considered the 21 candidates with miRNA* sequences as apple novel miRNAs and the remaining 21 without miRNA* sequences as apple miRNA candidates. Collectively, we term them apple-specific miRNAs. Of the 42 apple-specific miRNAs, 32 belong to the 21-nucleotide class of miRNAs and 10 to the 22-nucleotide class (Table 1). In general, the apple-specific miRNAs were much less abundant compared to the conserved miRNAs in all tissues examined. For example, only miRC1 displayed total read abundance above 20,000 RPM, while 33 of the 42 miRNA candidates yielded levels below 100 RPM (Table 1). This low level expression was further confirmed by RNA gel blot analysis showing that signal was detectable for only 18 of 42 apple-specific miRNAs (Figure 2). Almost all of the apple-specific miRNAs exhibited differential expression among tissues (Table 1 and Figure 2). For example, miRC1, miRC2, miRC5, miRC6, miRC9, miRC14, miRC15, miRC17, miRC18 and miRC20 showed preferential accumulation in either one or two tissues while miRC8 was exclusively expressed in leaf (Table 1 and Figure 2). As reported above for conserved and less-conserved miRNAs, RPM values for selected apple-specific miRNAs corresponded to relative signal intensity observed in RNA gel blots in some cases (miRC1 and miRC2), but several cases of divergence were observed as well. For example, miRC3 was the second-most abundant miRNA in fruit, miRC7 the most abundant in root and miRC10 was exclusively expressed in flower (Table 1), but RNA gel blots showed no or barely detectable signals for these three miRNAs in those tissues (Figure 2). As noted above for conserved and less-conserved miRNAs, RNA blotting revealed that the majority of the tested miRNAs were abundant in bark tissue from young seedlings, while very few were highly expressed in fruit (Figure 2).

**Table 1:** Novel or candidate miRNAs found in apple (excerpted^a^)

					Match		Normalized reads				
							
Name	miRNA sequence	**Contig**^**b**^	Len	Str	position	miRNA* sequence^c^	Leaf	Root	Flower	Fruit	Total
miRC1	ACAGGGAAGAGGTAGAGCATG	MDC006505.260	21	-	3,797	ATGCACTGCCTCTTCCCTGGC	1,091	23,246	0	12	24,349
miRC2	ACCTAGCTCTGATACCATGAA	MDC018599.370	21	+	9,369	TGTGGTATCAGGACTATGTTA	893	261	5,364	1,823	8,341
miRC3	CTACCGATGCCACTAAGTCCCA	MDC017130.228	22	+	6,809	GGACTTAGTAGCTCGGTGA	266	247	1,567	671	2,751
miRC4	TGTTATATTGTCAGATTGTCA	MDC019554.272	21	-	10,154	ACAGTCTGACAATATAACGTG	1,035	502	6	0	1,543
miRC5	AATGGAAGGGTAGGAAAGAAG	MDC006350.123	21	-	2,997	TCTTTCCTATCCCTCCCATTCC	1,021	324	144	0	1,490
miRC6a	TCCTCTTGGTGATCGCCCTGT	MDC009272.709	21	-	4,184	AGGGTGATTAACAAAGGGATG	359	279	336	319	1,294
miRC6b	TCCTCTTGGTGATCGCCCTGC	MDC006081.961	21	-	466	AGGGTGGTTACCAATGGGATG	122	218	0	46	385
miRC7	TTATACAGAGAAATCACGGTCG	MDC009778.59	22	-	4,735	ACCGTGTTTTTCTGTATAAAG	511	624	108	27	1,270
miRC8	AAGAGCGGGATGTGTAAAAGG	MDC001018.301	21	+	2,490	CTTTTACCTATCCCATTCTGT	248	0	0	0	248
miRC9	TCTGTCGTGGGTGAGATGGTGC	MDC011178.406	22	-	16,699	TTCATCTCTCCTCGACAGAAG	137	90	0	4	231
miRC10	GAATTCCTTCTCCTCTCCTTT	MDC026449.10	21	-	340	AGGAGGGAGAGAGGGTTTTAC	0	0	166	7	173
miRC11	CACCAATATCAACTTTATTTG	MDC005581.168	21	+	2,657	AATAAAGTTGATATTGGTGTG	6	12	44	38	100
miRC12	TCTGTCGAAGGTGAGATGGTGC	MDC003092.251	22	+	8,878	TTCATCCCTCCTCGACTGAAG	10	64	0	0	73
miRC13	ATCCAACGAAGCAGGAGCTGA	MDC009318.175	21	+	3,804	AGCTGCTGACTCGTTGGTTCA	0	0	18	42	60
miRC14	CGAACTTATTGCAACTAGCTT	MDC008558.180	21	+	6,507	CAAGCTAGTTGTAATAAGTTC	24	0	13	0	38
miRC15	AAAGTATCAAGGAGCGCAAAG	MDC014075.204	21	-	26,060	TTGCGTTCCACTGATTCTTTCG	8	8	9	2	27
miRC16	CATCTGGGTCGTTCAAATTTA	MDC009589.307	21	-	1,975	AATTTGAACGGCCCAGATGGG	10	12	0	0	22
miRC17	ATCATGCGATCCCTTCGGACG	MDC005391.194	21	+	11,429	TCCAAAGGGATCGCATTGATCT	11	0	0	0	11
miRC18	ATACTCATCGAATTTGTCATA	MDC012422.128	21	+	849	TGACAAATTGGATGAGTATTC	3	0	4	0	8
miRC19	TGGGATGTTGGTATGGTTCAA	MDC016463.170	21	-	5,187	GAGCCGTGCCAATATCACAGT	0	7	0	0	7
miRC20	TGAAGAGAAGAGCGTTGTTTGG	MDC001494.456	22	-	33,010	TGACAGCCTCTTCTTCTCATG	6	0	0	0	6
miRC21	ATCATTAACACTTAATAACGA	MDC006081.432	21	+	219	TTATTAAGTGTTAATGATTGG	0	0	0	2	2
miRC22	CCATATGTCCCTCCATATACT	MDC016302.308	21	+	5,368	No star found	0	0	72	8	80
miRC23	AATGATGATCAAACAACCCTT	MDC020884.221	21	+	4,086	No star found	0	0	46	2	48
miRC24	TGAACTTGGCTGAATGTGGACT	MDC001394.253	22	-	378	No star found	22	19	0	0	40
miRC25	TTTCGGAACCACTTACACCCA	MDC017130.228	21	+	9,764	No star found	0	0	24	0	24
miRC26	TCCCCAAAACCCCTCATTCCAA	MDC017130.228	22	+	9,967	No star found	0	0	15	0	15
miRC27	TTGCCAAAGGAGATCTGCTCAG	MDC019485.283	22	-	7,997	No star found	6	0	0	6	12
miRC28	TGCATTTGCACCTGCACTTGT	MDC007946.169	21	-	672	No star found	0	0	9	3	11
miRC29	CAAAGCTTTTAATATCAGTCGA	MDC018873.313	22	-	10,447	No star found	0	0	5	6	10
miRC30	TCCCTCAAGGGCTTCCAATATT	MDC004268.215	22	+	9,006	No star found	0	0	0	10	10
miRC31	TCCATAATTTTTCCAGATCAA	MDC005072.383	21	+	9,885	No star found	0	0	8	0	8
miRC32	TGGTTTGGTTGGAAAACGGCT	MDC006935.286	21	-	27,531	No star found	0	7	0	0	7
miRC33	AATTAGGCTGGCATTAGACAA	MDC009589.310	21	+	3,312	No star found	6	0	0	0	6
miRC34	TGGTGATAGGATAGTTGGAAG	MDC010150.221	21	-	29,453	No star found	6	0	0	0	6
miRC35	TACTGTTATAATGGCATTCCC	MDC001086.52	21	+	12,332	No star found	5	0	0	0	5
miRC36	CTCAATTTGAACGCGTGGCTA	MDC015454.116	21	+	3,773	No star found	0	5	0	0	5
miRC37	TGGCCTTGGTGGAAGAGATCC	MDC000614.265	21	-	5,670	No star found	0	4	0	0	4
miRC38	TGGGCCTGGTCAGGAGGATCC	MDC018501.179	21	-	2,535	No star found	0	4	0	0	4
miRC39	TTAAATACAAGCAGGAGCTCT	MDC011810.169	21	+	42,284	No star found	0	3	0	0	3
miRC40	CACCTGGGACTTGCAGCCATG	MDC009540.166	21	+	1,015	No star found	3	0	0	0	3
miRC41	CATCCGAATTACCAATAACTG	MDC009746.72	21	+	18,725	No star found	0	0	3	0	3
miRC42	ATAGATGGAAGCTACCAACCC	MDC013676.252	21	+	6,707	No star found	0	0	0	3	3

^a^Detailed information is listed in Table S5 in Additional file 1. ^b^Only one matched locus is shown in this table. Others are included in Table S5 in Additional file 1. ^c^The star sequence may have multiple variants. Only the best star sequence is shown in this table. Len, length; Str, strand.

### Targets of known and apple-specific miRNAs

To identify gene targets for the known (both conserved and less-conserved) and apple-specific miRNAs reported here, we performed degradome sequencing to generate a total of 21 million short reads representing 5' ends of uncapped, poly-adenylated RNAs. About 65% of the unique reads can be perfectly aligned to the apple transcriptome [52]. These reads were subsequently screened and analyzed with the software Cleaveland 2.0 [53,54]. A total of 118 targets that fell into 5 categories (0 to 4) were identified (Table 2; Table S6 in Additional file 1), with 62 targets for 14 of the 23 conserved, 38 for 5 of the 10 less-conserved, and 18 for 8 of the 42 apple-specific miRNAs or families (Table 2; Table S6 in Additional file 1).

**Table 2:** Example targets for apple miRNAs (or families)^a^

miRNA	Target	AS^b^	RN_reads^a ^(TPB)	Category^d^	Target gene annotation
**Conserved targets for conserved miRNAs**					
miR156	MDP0000146640	1	112.47	0	Squamosa promoter-binding-like protein
miR159	MDP0000147309	4.5	457.13	0	Transcription factor GAMYB
miR164	MDP0000121265	2.5	2,674.73	0	NAC domain-containing protein
miR165/166	MDP0000426630	3	212.24	0	Homeobox-leucine zipper protein
miR167	MDP0000137461	4	62.58	2	Auxin response factor
miR168	MDP0000161046	0	32.65	2	Argonaute protein
miR171	MDP0000151144	2.5	16.33	3	Scarecrow-like protein
miR172	MDP0000281079	3	212.24	0	Ethylene-responsive transcription factor RAP
miR319	MDP0000243495	3	16.33	2	Transcription factor TCP4
miR390	CN490861^e^				*MdTAS3*
miR393	MDP0000203334	2.5	2.72	3	Auxin signaling F-box protein
miR395	MDP0000121656	3.5	225.84	2	3'-Phosphoadenosine 5'-phosphosulfate synthase
miR396	MDP0000204597	4	32.65	0	Growth regulating factor (GRF)
tasiARFs	MDP0000179650	1	24.49	2	Auxin response factor
**Other non-conserved targets for conserved miRNAs**					
miR319	MDP0000296691	4.5	2.72	4	GDP-mannose 3,5-epimerase
miR396	MDP0000454027	4	5.44	4	IAA-amino acid hydrolase ILR1-like 6
miR399	MDP0000253476	4	21.77	3	Unknown
**Targets for other known miRNAs**					
miR2111	MDP0000416146	4	17.69	2	Unknown
miR3627	MDP0000941000	4	27.21	2	Amino acid transporter
miR535	MDP0000185769	4	27.21	3	Cysteine protease
miR828	MDP0000124555	1	69,205.65	0	MYB transcription factor
miR828	CN490819 (*MdTAS4*)	2.5	2,065.23	0	Non-coding mRNA
miR858	MDP0000140609	3	1,379.54	0	MYB transcription factor
miR858	MDP0000161125	4.5	351.01	0	Mate efflux family protein
miR858	MDP0000726382	4.5	2.72	3	Lipase family protein
**Targets for apple-specific miRNAs**					
miRC3	MDP0000485327	2	326.52	0	Unknown protein
miRC5	MDP0000863013	5	16.33	3	ARO4 (ARMADILLO REPEAT ONLY 4)
miRC6	MDP0000410000	5	16.33	2	Uncharacterized protein
miRC10	MDP0000294365	4	185.03	0	Protein kinase
miRC16	MDP0000737474	5	38.09	2	Uncharacterized protein
miRC25	MDP0000306273	4.5	10.88	2	Cytochrome P450 86B1
miRC29	MDP0000279462	4.5	13.60	2	Oligopeptide transporter 2
miRC42	MDP0000286564	5	27.21	2	Mitogen-activated protein kinase kinase 2

^a^A detailed list is included in Table S6 in Additional file 1. ^b^The alignment score (AS) threshold was set to 4.5 and 5 for known and apple-specific miRNAs, respectively. ^c^RN_reads, repeat normalized reads; TPB, transcripts per billion. ^d^Category 0: > 1 raw read at the position, abundance at position is equal to the maximum on the transcript, and there is only one maximum on the transcript. Category 1: > 1 raw read at the position, abundance at position is equal to the maximum on the transcript, and there is more than one maximum position on the transcript. Category 2: > 1 raw read at the position, abundance at position is less than the maximum but higher than the median for the transcript. Category 3: > 1 raw read at the position, abundance at position is equal to or less than the median for the transcript. Category 4: only 1 raw read at the position. ^e^Cleavage was predicted based on the distribution of tasiRNAs.

Among these targets for the conserved miRNA families, 13 fell into in category 0, which represented the most abundant degradome tags corresponding to the cleavage site and matching cognate transcripts, and 25 of them into category 2, whose cleavage abundance was higher than the median but below the maximum. The number of identified gene targets varied for different miRNAs, ranging from one to nine (Table 2; Table S6 in Additional file 1), but those miRNAs that targeted members of a gene family usually had more targets. For example, miR156 could target nine members of the squamosa promoter-binding-like protein family, and miR167 targeted six members of the auxin response factor (ARF) family (Table 2; Table S6 in Additional file 1). Although most of the genes (54 of 62) identified were the conserved targets for these miRNAs across a wide range of plant species, a few of them (8 of 62) had not been reported in other species. For example, miR319, which is known to target *TCP4 *in other species, was found to target two genes coding for GDP-mannose 3,5-epimerase. Similarly, miR396, which exclusively targeted several members of the growth regulating factor (*GRF*) gene family in plants also targeted five IAA-amino acid hydrolase genes, three replicate factor C subunit 1 genes and one TIR-NB-LRR resistance gene. It was noted that a few identified apple-specific gene targets fell into category 4, which represents a low confidence group and might need to be further validated experimentally. Of the 38 targets identified for five less-conserved miRNAs or families, a single target was found for miR2111, miR3627, and miR535 (Table 2; Table S6 in Additional file 1). The remaining 35 targets identified were shared by miR828 and miR858, with the former targeting four *MYB *genes and *MdTAS4 *and the latter targeting up to 30 genes, including 24 coding for MYB factors, 2 coding for mate efflux proteins and 3 coding for lipases (Table 2; Table S6 in Additional file 1). miR828 and miR858 have been shown to target *MYB*s in other species but their target number was very limited [36,55]. Finding an unusually large number of *MYB *targets for miR828 and miR858 suggests that they gained more diverse and broad regulatory roles in apple.

Gene targets were also identified for eight apple-specific miRNAs. Of the 18 gene targets identified, two belonged to category 0 and seven to category 2, while the remaining were classified into category 3 or 4 (Table 2; Table S6 in Additional file 1). Most of the apple-specific miRNAs, unlike their conserved counterparts, had relatively fewer gene targets with a higher alignment score. The apple-specific miRNAs, like conserved ones, targeted genes with diverse functions. For example, miRC5 targeted a gene coding for ARO4 protein while miRC42 targeted a gene encoding mitogen-activated protein kinase 2. miRC25 and miRC29 each targeted two members of gene families that code for cytochrome P450 and oligopeptide transporter 2, respectively. Further, miRC10 targeted up to six members of the translation initiation factor 2 subunit beta gene family and one protein kinase gene. Hence, these apple-specific miRNAs may be involved in regulation of an array of metabolic and biological processes and signaling pathways.

### Three miRNAs target an unexpectedly large number of *MYB *genes in apple

The *MYB *gene family represents one of the largest families in plants, and some of its members are regulated by miRNAs [56]. In *Arabidopsis*, miR159, miR828 and miR858 were either predicted or confirmed to target at least 13 *MYB *genes [56,57]. Our degradome analysis confirmed they collectively targeted 29 *MYB*s (Table 2; Table S6 in Additional file 1, and Figure S2a, b in Additional file 2), which raised a question of how many genes these miRNAs actually targeted because the degradome analysis in this study identified less than 40% of the targets for the conserved miRNAs and an even lower percentage for the less-conserved and apple-specific miRNAs. To address the possibility that some *MYB *gene targets were missed during degradome analysis, possibly due to inactive or low levels of target gene expression in the plant tissues analyzed, we performed target prediction analysis in over 400 putative apple *MYB*s and identified an additional 8, 15 and 42 *MYB *genes with a cleavage-favorable alignment score (≤5) for miR159, miR828 and miR858, respectively. Thus, a total of nine *MYB*s for miR159, 19 for miR828 and 66 for miR858 were found, bringing the total number of *MYB*s potentially regulated by these miRNAs to 81 (Figure 3a; Table S7 in Additional file 1). We also found that miR858 shared 11 targets with miR828 and two with miR159 (Figure 3a; Table S7 in Additional file 1), but no common target was identified for miR828 and miR159.

MYB proteins are divided into four classes, 1R, 2R (R2R3), 3R (R1R2R3) and 4R, depending on the number of adjacent repeats homologous to R1, R2 and R3 in the animal c-Myb [56], but most MYBs in plants belong to the R2R3 class [58,59], many of which share a very similar genomic organization and protein structure with a conserved region at the 5' end and a divergent one at the 3' end (Figure 3b). Out of the 81 *MYB *genes that we confirmed or predicted as miRNA targets, 67 belonged to the R2R3 class. The miR159 target site was found to locate in the sequence-divergent region, while the miR858 and miR828 target sites both mapped to a 55-nucleotide region in the conserved coding region upstream of the divergent region, and the two sites were separated by a 12-nucleotide fragment with the position of the miR858 target site at the 5' end and that of miR828 at the 3' end (Figure 3b). The dual cleavage by miR858 and miR828 was confirmed in one of the targeted *MYB*s (MDP0000124555) by RNA ligation-mediated 5' rapid amplification of cDNA ends (RLM-5'-RACE) analysis (Figure 3b). Strikingly, this 55-nucleotide fragment encompassing the miR858 and miR828 targeted sequences and 12-nucleotide spacer was found to be highly conserved across a wide range of dicots and monocots (Figure 3b). The finding that miR828 and miR858 co-targeted a group of *MYB *genes prompted us to examine whether they were co-expressed or differentially regulated among apple tissues. Figures 1b and 3c show that miR828 and miR858 exhibit a distinct expression pattern that was generally corroborated by both RNA gel blots and RNA sequencing. miR828 was specifically expressed in flower while miR858 accumulated in all tissues tested, but was found to be most abundant in mature fruit (Figure 3c), suggesting that miR828 and miR858 differentially regulated their co-targeted *MYB*s in different tissues.

### Potential functions of the miRNA-targeted *MYB*s in apple

In *Arabidopsis*, the R2R3 *MYB *gene family comprises 25 subgroups and includes many members that have been functionally characterized and conserved between divergent species [56-58]. These previous characterizations could be instrumental for deciphering the function of apple *MYB*s and the possible regulatory roles of these *MYB*-targeting miRNAs in apple. We performed phylogenetic analysis for those 81 miRNA-targeted apple *MYB*s with *Arabidopsis *R2R3 *MYB*s to investigate their potential functions (Figure 3d). Six of the nine miR159-targeted *MYB*s were placed into *MYB *subgroup 18 involved in anther and pollen development, while the remaining three were close to subgroup 25, which is associated with embryogenesis in *Arabidopsis *(Figure 3d). Hence, miR159 may regulate male organ and embryo development and growth in apple. The 19 miR828-targeted *MYB*s were related to three subgroups: S6, S7 and S15 (Figure 3d). Subgroups S6 and S7 were shown to directly or indirectly control anthocyanin biosynthesis in plant tissues, while S15 *MYB*s are involved in regulating trichome initiation and root hair patterning. Notably, most of the miR828-targeted *MYB*s are linked with primary and secondary metabolism related to anthocyanin production and color development. The 66 *MYB*s targeted by miR858 represent at least 14 subgroups shown to regulate diverse biological processes and metabolism pathways relevant to cell wall formation, lignification, anthocyanin biosynthesis, cell fate and identity, plant development and response to biotic and abiotic stresses in *Arabidopsis*. Nine of the ten *MYB*s co-targeted by miR828 and miR858 cluster together within subgroup 5, which is involved in the regulation of proanthocyanidin biosynthesis (Figure 3d). Thus, the roles of miR858-mediated regulation of *MYB*s in apple are predicted to be much broader than those for either miR828 or miR159. Of the 81 *MYB*s analyzed, the 29 *MYB*s confirmed as targets by degradome analysis fell into at least seven subgroups (S6, S5, S9, S14, S15, S18 and S20), with the majority of the confirmed *MYB*s clustered with the S5 and S6 groups, which are primarily involved in anthocyanin biosynthesis (Figure 3d).

### The co-targeting sequence of miR828 and miR858 is located in the region encoding the conserved R3 repeat domain of MYB proteins

**Figure 4:**
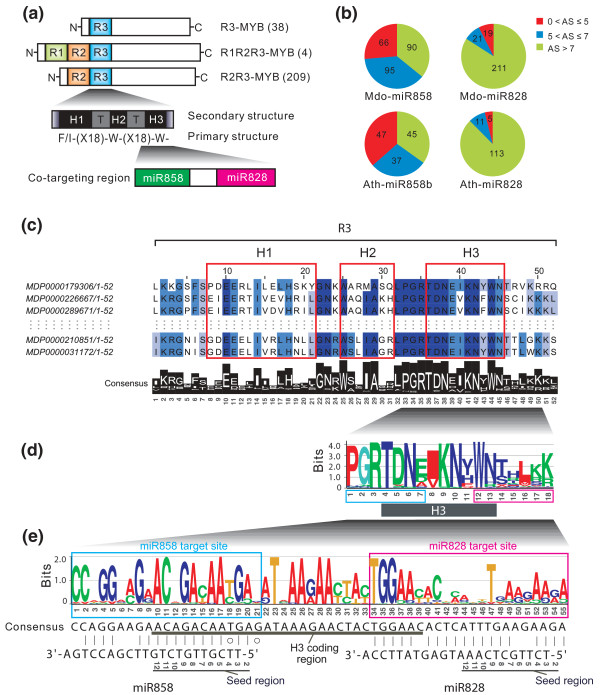
**The co-targeting sequence of miR858 and miR828 is located in the region encoding the R3 repeat domain of MYB proteins**. **(a) **Schematic diagram of MYB proteins, and the location of the co-targeting region of miR828 and miR858. The MYBs containing a single or multiple R domains (R1, R2 and R3) are indicated. H, helix; T, turn; W, tryptophan; X, amino acid. **(b) **Distribution of alignment score (AS) between miRNAs (miR828 and miR858) and their target sites among 251 apple (upper pie charts) and 129 *Arabidopsis MYB *genes (lower pie charts). AS categories: ≤5, cleavage-favorable; 5 < AS ≤ 7, less cleavage-favorable; > 7, cleavage-unfavorable. **(c) **Conservation of the H3 helix and its flanking sequences among 251 apple MYB proteins. The conservation of amino acid residues in each position is denoted with various colors: dark color indicates a high level; light color indicates a low level of conservation. **(d) **Conservation of 18 amino acid residues encoded by the miR828 and miR858 co-targeting region. The bit value representing the conservation in each position is indicated. **(e) **Conservation of the 55-nucleotide co-targeting region in 251 apple *MYB *genes. The pairing between the consensus sequence and each miRNA is illustrated. miRNA target sites, the H3 coding region and the miRNA seed region are also marked.

That miR828 and miR858 targeted substantially different numbers of *MYB *genes despite the adjacent location of their target sites prompted us to examine conservation profiles of their target sequences at both the amino acid and nucleotide levels (Figure 4). We found that the18 amino acid polypeptide encoded by the 55-nucleotide sequence that bears both miR828 and miR858 target sites was located in the conserved R3 DNA binding domain of MYB factors (Figure 4a). Homology searching against the whole apple proteome using the18 amino acid polypeptide obtained a total of 251 apple MYB factors containing this signature sequence, with 209 belonging to the R2R3 group and 4 and 38 belonging to the R1R2R3 and R3 groups, respectively (Figure 4a). The R3 domain consists of three α-helices (Figure 4a, c), and the third helix (H3) in each MYB repeat domain makes direct contact with its DNA target with the assistance of the first and second helices (H1 and H2) in basic helix-loop-helix (bHLH) motif folding (Figure 4a, c) [60,61]. Among the three helices in the R3 domain, the H3 helix that encompasses ten amino acid residues was most conserved among all MYB factors analyzed (Figures 4c; Figure S3a in Additional file 2). Of the 18 amino acid residues, the first seven (1 to 7) encoded by the 21-nucleotide miR858 target site were located in the highly conserved region covering three amino acid residues upstream and four amino acid residues at the 5' end of H3 while most of the last seven (12 to 18) encoded by the miR828 target site were located in the much less conserved region downstream of H3 (Figure 4c, d). Similar homology searching in *Arabidopsis *found that 129 MYBs, including 124 R2R3 and 5 R1R2R3 MYBs, bear a similar signature sequence in the R3 domain (Figure S3b in Additional file 2). Correspondingly, the miR858 target site was found to be more conserved than the miR828 target site at the nucleotide level in both apple and *Arabidopsis *(Figure 4e; Figure S3b in Additional file 2). This difference was particularly pronounced in a region (positions 10 to 20 in the miR858 target site, and 44 to 54 in the miR828 target site) that specifically pairs with the miRNA seed region (positions 2 to 12) (Figure 4e; Figure S3b in Additional file 2). Since pairing between the miRNA seed region and corresponding target site is critical for miRNA cleavage [62], the level of sequence conservation in this region could impact miR828- and miR858-targeted *MYB *populations.

Based on alignment scores ≤5, 66 and 19 apple *MYB*s were identified to be targeted by miR858 and miR828, respectively (Figure 3a). Given that many target sites with high alignment scores > 5 have been proven to be cleavable [63,64], the actual number of MYB targets is likely to be larger than what we reported in Figure 3a. Therefore, we further analyzed the alignment score distribution profiles for these two miRNAs among all the 251 apple *MYB*s, and found 95 of the 251 *MYB*s with a less cleavage-favorable alignment score (> 5 and ≤7) and 90 with a cleavage-unfavorable alignment score (> 7) with miR858 (Figure 4b, top). In contrast, 211 of the 251 *MYB*s showed a cleavage-unfavorable alignment score (> 7) with miR828 while only a very small portion of them had a cleavage-favorable or less cleavage-favorable alignment score (≤7) (Figure 4b, top). A similar pattern was observed among 129 *Arabidopsis MYB*s (Figure 4b, bottom). These results imply that the targeting capacity of miR858 and miR828 in apple and *Arabidopsis *might be even broader than those reported in Figure 3a, especially for miR858.

### tasiRNA biogenesis pathways with unique features evolved in apple

**Figure 5:**
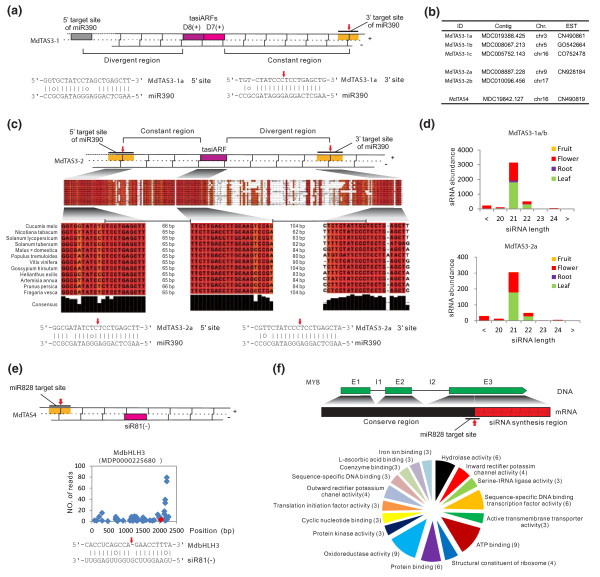
**Apple miRNA-*TAS *pathways and *MYB*-derived tasiRNA-cascaded regulatory network**. **(a) **Schematic diagram for siRNA biogenesis along *MdTAS3-1 *family transcripts. Two conserved tasiARFs are flanked by a length-constant region at the 3' end and a divergent region at the 5' end. The parings between miR390 and its target sites are illustrated below. **(b) **List of apple *TAS *genes. **(c) **Schematic diagram of the siRNA biogenesis of *MdTAS3-2 *family transcripts and their conservation among plant species, including *Cucumis melo *(AM737743), *Nicotiana tabacum *(AM791738), *Solanum lycopersicum *(BE459870), *Solanum tuberosum *(BQ514736), *Malus × domestica *(CN928184), *Populus tremuloides *(DN500355), *Vitis vinifera *(DT023634), *Gossypium hirsutum *(DW502659), *Helianthus exilis *(EE660948), *Artemisia annua *(GW328601), *Prunus persica *(scaffold_3:16167014..16167205), and *Fragaria vesca *(scf0512980:217347..217557). The pairings between miR390 and its target sites are illustrated below. **(d) **Expression profiles for tasiRNAs in *MdTAS3-1a/b *and *MdTAS3-2a*. **(e) **Schematic diagram of the siRNA biogenesis of *MdTAS4 *and confirmation of a new gene target for *TAS4*-derived siR81(-) in apple. The miR828 cleavage of *MdTAS4 *transcripts and generation of the conserved *MdTAS4*-siR81(-) are marked on the transcript. The confirmed cleavage of the transcript of a gene coding for a bHLH3 factor by degradome is presented as a T-plot, and sequence pairing with cleavage site marked is shown below. **(f) **Location of the siRNA-biogenesis region among the ten miR828-targeted *MYB*s, and potential gene targeted by *MYB*-derived siRNAs. The number of genes or gene families targeted by siRNAs is indicated inside parentheses.

To date, only four *TAS *families (*AtTAS1-4*) and three miRNAs (miR173, miR828, and miR390) that target *TAS *transcripts and trigger tasiRNA production have been reported and well characterized in *Arabidopsis *[5,18-20,30]. Both miR390 and miR828 were identified in apple (Figure 1a, b), and showed highest expression specifically in flower as detected by RNA blot and RNA sequencing methods (Figures 1a, b and 3C). A *TAS4 *homolog, *MdTAS4*, was found in apple (Figure 5e), and degradome analysis showed that miR828 cleaved *MdTAS4 *(Figure S4E in Additional file 2). Sequencing data showed that abundant 21-nucleotide sRNAs were produced along the 3' cleaved *MdTAS4 *transcript, and most of those sRNAs belonged to the first (miR828 target site) and second register while some of them fell into the 12th register (Figure S4E in Additional file 2). Analysis of siRNA abundance in four libraries showed that *MdTAS4-*derived tasiRNAs primarily accumulated in flower tissues (Figure S4E in Additional file 2), which is in agreement with the flower-biased expression of miR828 in apple (Figure 3c). In *Arabidopsis*, siR81(-), one of the *AtTAS4*-derived siRNAs, was shown to target *AtMYB75*, *AtMYB90 *and *AtMYB113*, which are associated with anthocyanin biosynthesis [30,65]. Our analysis also predicted that apple *TAS4*-siR81(-) potentially targeted at least three *MYB *homologs (data not shown), and degradome data confirmed that apple *TAS4*-siR81(-) targeted an additional gene (MDP0000225680) coding for a bHLH transcription factor (Figure 5e), which is also involved in the regulation of anthocyanin biosynthesis in apple [66]. Thus, apple *TAS4*-siR81(-) is likely to target both *MYB *and *bHLH *genes.

The apple genome was rich in *TAS3 *homologs, and at least two *TAS3 *gene families, termed *MdTAS3-1 *and *MdTAS3-2*, were identified. *MdTAS3-1 *has at least three homologs (Figure 5b). *MdTAS3-1a *and *MdTAS3-1b *share about 98% sequence identity, but each share only 80% sequence identity with *MdTAS3-1c*. Our deep sequence data show that both *MdTAS3-1a/b *and *MdTAS3-1c *have two miR390 cleavage sites flanking phased-tasiRNA generation regions (Figure 5a). The 5' target site bears a conserved mismatch in the tenth position, as its counterpart does in *Arabidopsis*, and should be non-cleavable, while the siRNA distribution data suggest that the 3' site could be cleavable and likely sets the phasing for the production of 21-nucleotide tasiRNAs (Figure S4a, b in Additional file 2). The two conserved tasiARFs are flanked by a constant tasiRNA-generation region at the 3' end and a divergent region at the 5' end (Figure 5a). These features are consistent with canonical features characterized for *AtTAS3 *in *Arabidopsis *[20]. Interestingly, tasiRNAs from each member displayed leaf- and flower-biased accumulation despite the nearly exclusive expression of miR390 in flower (Figures 1a, 3c and 5d; Figure S4f in Additional file 2).

The *MdTAS3-2 *family exhibited similar and distinct features relative to the *MdTAS3-1 *family. The two *MdTAS3-2 *homologs, *MdTAS3-2a *and *MdTAS3-2b*, are relatively short compared to the *MdTAS3-1 *family and share about 85% sequence identity (Figure 5b). Like the *MdTAS3-1 *family, both *MdTAS3-2a *and *MdTAS3-2b *transcripts have two miR390 cleavage sites flanking an approximate 190-nucleotide region for 21-nucleotide phased siRNA production (Figure S4c, d in Additional file 2). In contrast to *MdTAS3-1*, *MdTAS3-2 *family transcripts encode only one tasiARF, and there was no mismatch in the tenth position of the 5' miR390 target site (Figure 5c). Moreover, these short *TAS3 *genes are absent in *Arabidopsis *but conserved in many dicots sharing the presence of the dual miR390 target sites and production of a single tasiARF (Figure 5c). Unlike *MdTAS3-1*, the regions flanking the *MdTAS3-2 *tasiARF are orientationally reversed, with a constant region at the 5' end and a divergent region at the 3' end (Figure 5c). Based on the siRNA distribution pattern, the 3' site of miR390 is predicted to set the phase for siRNA generation (Figure S4c, d in Additional file 2). Similar to *MdTAS3-1*, *MdTAS3-2*-derived tasiRNAs also preferentially accumulated in both leaf and flower tissues but with much less abundance (Figure 5d; Figure S4f in Additional file 2).

Earlier studies showed that *Arabidopsis TAS3*-derived tasiARFs directly target several *ARF *genes [67], which were proposed to act as suppressors in the auxin signaling pathway [68]. Our degradome data show that they guide the cleavage of at least three apple *ARF *transcripts (Figure S2c in Additional file 2). Homologous sequence alignment revealed that the cleaved apple *ARFs *are closely related to *AtARF2*, *AtARF3 *and *AtARF4*.

### miR828-activated, *MYB *transcript-derived siRNAs and their gene targets

The possibility that miR828-cleaved *TAS4 *RNA fragments could be channeled into tasiRNA biogenesis [30,65] led us to examine whether all miR828-cleaved *MYB *transcripts are also subjected to tasiRNA biogenesis. With direct searching against small sequencing libraries, we were able to map a large number of sRNA reads to the coding regions of ten miR828-targeted *MYB*s. These *MYB*s share similar genomic organization with the location of the miR828 target site in the third exon just before the divergent region where siRNA biogenesis occurred (Figure 5f). Further analysis showed that the generated siRNAs were in phase with the miR828 cleavage site, and the siRNA generation pattern varied among the ten *MYB*s (Figure S5 in Additional file 2). Interestingly, despite the apparent flower-biased expression of miR828 (Figure 3c), distinct patterns of accumulation of phased siRNAs were observed among the ten miR828-targeted *MYB*s (Figure S5 in Additional file 2). We found that a total of more than 100 phased, sequence-distinct 21-nucleotide siRNA species were produced from the cleaved 3' transcripts of these *MYB*s (Figure 5f). To ascertain whether these siRNAs were able to guide the cleavage of other gene transcripts, we carried out degradome analysis with a stringent alignment score (≤4.5) and found that they could potentially target as many as 77 genes, including six *MYB *genes, and a diverse array of other genes encoding proteins such as potassium transporters, protein kinases, hydrolases, oxidoreductases, transcription factors and DNA-, protein- and ion-binding proteins (Figure 5f; Table S8 in Additional file 1).

## Discussion

### Apple miRNAs with conserved as well as new gene targets

A recent study reported 16 conserved and less-conserved miRNAs in apple based on bioinformatics prediction using EST sequences [47], which is far more limited compared to those identified in other plant species [30,35,36]. In this study, we employed deep sequencing and computational analyses to identify 33 known (23 conserved and 10 less-conserved) miRNA families and 42 apple-specific (21 novel and 21 candidate) miRNAs (Figure 1 and Table 1; Table S3 in Additional file 1), which provides, to date, the most comprehensive list of identified miRNAs in apple. The majority of these miRNAs displayed tissue-specific expression (Figure 1), which is consistent with a general scenario in which miRNAs are differentially regulated in fruit trees [36,38,46,47] and other species [30,35,69], although additional work is needed to resolve examples of apparently divergent results from RNA gel blotting versus sRNA sequencing, as noted in this report and elsewhere [30,36]. It is known that miRNAs are involved in regulation of leaf morphology and polarity, lateral root formation, hormone signaling, phase transition, flowering time, floral organ identity and reproduction, anthocyanin production and stress and pathogen response [8,65,70,71]. In apple, we have identified a total of 100 gene targets for 19 of the 33 known miRNAs using degradome analysis (Table 2; Table S6 in Additional file 1), and the majority of these targets are conserved in plant species, indicating broad conservation of the known miRNA regulatory roles in plants. However, a few of the known miRNAs, including miR319 and miR396, were found to target additional genes in apple that have not been previously reported, while others like miR828 and miR858 target an unexpectedly large number of *MYB *genes. Hence, while these known miRNAs conserve their gene targets, they also appear to have an expanded target gene population in apple.

Although many newly evolved miRNAs that may exhibit weak expression, imperfect processing and lack of targets are believed to serve no biological function, many of them have been shown to target and regulate specific genes or gene families in various species [36,64,72]. Eight of the 42 apple-specific miRNAs or candidates were also found to target specific genes, implicating these miRNAs in the control of signal transduction cascades, secondary metabolism and protein translation (Table 2; Table S6 in Additional file 1). Our inability to detect gene targets for the remaining apple-specific miRNAs or candidates may be due to a low level of expression or the stress- or developmentally inducible nature of their target genes.

### Apple *TAS *gene families with unique features and target specificity

In *Arabidopsis*, four *TAS *families targeted by three miRNAs, including miR173, miR390 and miR828, have been characterized [5,17-20,30,63]. The miR390-*TAS3 *pathway is highly conserved in the plant kingdom [20], and the miR828-*TAS4 *pathway is widely represented in dicot species [65], but no miR173-*TAS1*/*TAS2 *pathway has been found in other species besides *Arabidopsis*. In this study, we showed that apple conserved the miR390-*TAS3 *and miR828-*TAS4 *pathways with expanded features. Apple possesses an additional *MdTAS3-2 *family that comprises two loci and transcribes short mRNA species with distinct structural organization of the siRNA generation region, which bears only one characteristic functional tasiARF, instead of the two in *MdTAS3-1 *transcripts (Figure 5c). The *TAS3-2 *family is not present in *Arabidopsis *but is widely conserved in many other dicot species (Figure 5c) [73]. Despite an extra *TAS3 *family in apple, the derived tasiARFs were found to target similar *ARF *genes homologous to the *Arabidopsis *genes *AtARF2*, *AtARF3 *and *AtARF4 *(Figure S2c in Additional file 2), which negatively regulate auxin signaling [68]. Whether these genes are differentially targeted by *MdTAS3-1 *or *MdTAS3-2 *tasiARFs is difficult to determine. Nevertheless, the existence of more *TAS3 *genes with distinct expression patterns could enable the auxin signal to be fine-tuned within a specific cell, tissue or developmental context.

One interesting feature of the apple miR828-*TAS4 *pathway is that its derived tasiRNA, *MdTAS4*-siR81(-), targets an additional gene. In *Arabidopsis*, *AtTAS4*-siR81(-), a conserved siRNA derived from the phased siRNA production of *AtTAS4*, is shown to target at least three *MYB*s that positively regulate anthocyanin accumulation in response to environmental stresses [30,65,74]. In apple, besides the three predictable *MYB*s, *MdTAS4*-siR81(-) also targets a bHLH transcription factor (*MdbHLH3*) that interacts with *MdMYB10 *(MDP0000259614) to regulate anthocyanin biosynthesis in apple [66].

### Unique miR828-activated, siRNA-cascaded gene regulatory network and its potential biological function

One of the interesting findings in this study is that miR828 potentially targets up to 19 *MYB*s in apple, 10 of which are subjected to siRNA biogenesis, with production of over 100 diverse siRNA species from the diverged region of *MYB*s (Figure 5f). In *Arabidopsis*, miR828, which indirectly targets *AtMYB113 *through *AtTAS4*-siR81(-), also directly targets *AtMYB113 *[65]. Up-regulation of miR828, *AtTAS4 *and *AtTAS4*-siR81(-) is correlated with that of their three direct or indirect targets (*PAP1/AtMYB75*, *AtMYB90 *and *AtMYB113*) under phosphate (Pi) and nitrogen deficiency conditions [74], which appears to contradict the anticipated negative role of miR828 in regulation of anthocyanin production. Interestingly, the elevated expression of *PAP1/AtMYB75 *(and possibly *AtMYB90 *and *AtMYB113*) induces miR828 and *AtTAS4 *expression, presumably through binding of these *MYB*s to the 5' *cis*-elements in *MIR828 *and *AtTAS4 *promoter regions [74]. Such autoregulatory feedback was proposed to maintain proper anthocyanin production under stress conditions [65,74]. Conceivably, the miR828-activated siRNA biogenesis in seven of the ten targeted *MYB*s that relate to anthocyanin accumulation in apple would reinforce this feedback regulation to ensure proper color appearance in a specific tissue or apple fruit during development. However, identification of over 70 genes as targets for the *MYB*-derived siRNAs suggests that they may function beyond the feedback regulation of anthocyanin accumulation (Figure 5f; Table S8 in Additional file 1). The identified targeted genes are predicted to code for proteins regulating diverse functions ranging from hydrolase, oxidoreductase and kinase activities, and iron transport to DNA-, ATP-, and co-enzyme-binding activities (Figure 5f; Table S8 in Additional file 1), suggesting that this miRNA-activated, *MYB*-dependent and siRNA-cascaded gene regulation might orchestrate major physiological or biochemical or secondary metabolism switches associated with anthocyanin production and the pigmentation process.

### miRNAs as master regulators to regulate a large number of *MYB*s through targeting of conserved sequences

The finding that three miRNAs potentially target up to 81 different *MYB*s indicates that miRNAs can, like transcription factors, serve as master regulators to modulate expression and function of a large number of genes in plants. This unique regulatory network is primarily based on the high degree of sequence pairing between miRNAs and their targeting sites as well as the availability of the miRNA target sites among the *MYB *population. *MYB *genes typically share a conserved 5' region and diverge at their 3' end [56]. Conceivably, the miRNA-targeted sites residing in highly conserved functional domains would be necessarily preserved among the *MYB *population relative to those located in the divergent region. Consistent with this prediction, miR828 and miR858 target sites, which overlap the conserved R3 region, are found in more *MYB*s than the miR159 target site located in the divergent region (Figure 3a). Similarly, the miR858 target site, which overlaps the more highly conserved 5' end of the H3 domain, is conserved in more *MYB*s than the miR828 site, which overlaps the less conserved 3' end of the H3 domain (Figure 4c-4e). Thus, sequence conservation and divergence of the miRNA target sites could directly impact the miRNA targeted gene population within a gene family.

Although the footprint of the 55-nucleotide sequence encompassing both miR828 and miR858 target sites is detected in dicot and monocot species (Figure 3b), miR828 and miR858 emerged only in dicot species [41], indicating that miR828- and miR858-mediated regulation of *MYB *genes is a feature of dicot species, consistent with our finding that miR828 and miR858 target a large number of *MYB*s in both apple and *Arabidopsis *(Figure 3a). It is not clear why this regulatory network specifically occurs in dicots while large *MYB *families exist in monocots as well [75]. Currently, it is known that *MYB*s are differentially regulated by transcription factors [76] as well as a variety of post-translation interactions or modifications. Our new discoveries related to miRNA-mediated regulation of a multitude of *MYB*s strengthens our understanding of how apple and other dicot species integrate transcriptional, post-transcriptional and post-translational regulatory mechanisms to achieve exquisite spatio-temporal regulation of each member of the *MYB *family.

Intriguingly, miR858 was found to co-target 11 *MYB*s with miR828 and two *MYB*s with miR159 (Figure 3a), raising the question of whether the convergence of two miRNAs upon the same *MYB *genes is an evolutionary coincidence or conveys some biological significance. The latter possibility is favored by the fact that 10 out of 11 *MYB*s co-targeted by miR828 and miR858 are related to regulation of anthocyanin biosynthesis. miR828 and miR858 may either redundantly reinforce each other's silencing function or differentially regulate anthocyanin accumulation in various apple tissues. The detection of differential expression of miR828 and miR858 among various tissues appears to support their different regulatory roles (Figure 3c).

The targeting of multiple members of gene families by one or a few miRNAs is not unique to the *MYB *family. Recently, a similar regulatory strategy was reported for *NB-LRR *defense genes in *Medicago*, where three miRNAs collectively target over 70 *NB-LRR *loci [77]. Since plant genomes have evolved many large gene families with unique sequence conservation features, such regulatory strategies could be conceivably adopted by various species to modulate large groups of genes. Further characterization of this mechanism for regulating multi-gene families among different species could provide insight regarding both their evolution and function.

## Conclusions

We carried out extensive characterization of miRNAs, their targets and expression in apple and provide a comprehensive list of miRNAs identified. We show that apple conserves and has evolved a variety of miRNAs with distinct expression patterns, and these miRNAs target dozens of apple genes with a wide range of functions. The discovery of an additional short *MdTAS3 *family suggests that miR390 and tasiARFs may play more complicated roles in the auxin signaling pathway. More importantly, we reveal the existence of two similar but distinct regulatory networks in apple: direct miRNA targeting of a large number of *MYB*s and miR828-activated and *MYB*-derived siRNA-cascaded targeting of 77 genes primarily outside the *MYB *family, which has not yet been reported in other species.

## Materials and methods

### Plant material

*Malus × domestica *of 'Golden delicious', grafted on 'M.111' rootstock, was selected in an orchard located in the Alson H Smith Agricultural Research and Extension Center for tissue collection of leaf, flower and fruits. Root and bark tissues were collected from rapidly growing, two-year-old seedlings. Fruits were harvested at 15 and 120 days after anthesis (DAA).

### RNA preparation and small RNA sequencing

Total RNA from different tissues was extracted using the Plant RNA Purification Reagent (Invitrogen, Grand Island, NY, USA). sRNA quantity and quality was evaluated by the Agilent 2100 Bioanalyzer. RNA samples of RNA integrity number (RIN) above 8 were sent to BGI (Hong Kong, China) for sRNA and degradome sequencing using standard protocols on the SOLID sequencing system or Illumina Hiseq 2000 platform.

### Small RNA data analysis

Small RNA libraries were constructed and sequenced for four apple tissues. The GenBank Gene Expression Omnibus (GEO) accession number for the sequencing data is GSE36065. All the sequencing data were first processed by removing the 3' adaptor sequence using CLC Genomic Workbench 4.9 (CLC bio, Aarhus, Denmark). Any sequences without adaptor matches were excluded from further analyses. Reads homologous to non-coding RNAs and conserved miRNAs were removed by BLATN alignment against the Rfam 10 [78] and mature miRNAs collected in miRBase (release 17) [79], allowing up to two mismatches. The remaining sRNAs were subjected to new miRNA identification. Read mapping was conducted using Bowtie [80], and Vienna RNA package [49] was used for the secondary structure prediction of sRNAs. Only those sRNAs (20- to 22-nucleotide) with a good stem-loop structure (no more than four mismatches, and no more than one central bulge) and a miRNA/miRNA* pair accounting for more than 75% reads matching to the precursor locus were considered as potential miRNAs (Additional files 3 and 4). Detailed screening critera were set up according to Meyers *et al. *[51]. The total number of reads perfectly matching the apple genome in a given library was used for the normalization of read abundance, which was denoted as RPM (reads per million reads). Apple genome sequences were retrieved from the Genome Database for Rosaceae [52]. The R package was used for the construction of heat maps.

### Multiple alignment, phylogenetic analysis and Gene Ontology annotation

Multiple alignment was conducted using CLUSTAL X2, with the coloration based on the residue identity (above 60%) [81]. All the apple *MYB *targets for miR828, miR858, and miR159 were predicted by Targetfinder 1.6 with the alignment score no more than 5. Amino acid sequences of 126 R2R3 and 5 R1R2R3 MYB factors in *Arabidopsis *were retrieved from TAIR [82]and the phylogenetic tree was inferred using the neighbor-joining method and 1,000 bootstraps with putative full-length sequences using CLUSTAL X2 [81]. The subgroup and function annotation were designated according to Dubos *et al. *[56]. Gene Ontology annotation for the target genes of the *MYB*-derived siRNAs was performed using Blast2GO [83] with the default settings.

The *MYB*s, including 251 from apple and 129 from *Arabidopsis*, were retrieved by using the conserved 18 amino acid sequence corresponding to the co-targeting region (PGRTDNEIKNYWNTHLKK) to blast against the Apple Genome V1.0 predicted peptides [52] and the *Arabidopsis *TAIR10 proteins [82] with an e-value of 100. The classification of MYB subfamilies (R3, R2R3, R1R2R3) was based on the quantity of conserved R-repeats identified by NCBI Conserved Domain Search [84]. The consensus nucleotide sequences of the co-targeting region were obtained by counting the most frequently appearing residue at the corresponding position. The alignment score of each target site was calculated according to the scoring algorithm established by Allen *et al. *[18]: mismatches and single-nucleotide bulges or gaps were assessed by a penalty of 1 while GU base pairs were assessed by a penalty of 0.5; and the penalty score from mismatches, bulges, gaps and GU pairs for positions 2 through 13 was doubled. Sequence logos were produced by GENIO/logo [85].

### RNA gel blot

For RNA gel blot analysis, 25 to 50 μg of total RNA from apple leaf, bark, root, flower, young fruit and mature fruit was separated on 15% denaturing polyacrylamide gel and transferred to Amersham HybondTM-NX membranes (GE Healthcare, Waukesha, WI, USA). RNA was cross-linked using EDC (N-(3-dimethylaminopropyl)-N'-ethylcarbodiimide hydrochloride (Sigma, St Louis, MO, USA). The probes of 21-nucleotide DNA oligonucleotides (Table S9 in Additional file 1) that are reverse complementary to apple-specific miRNA candidates were labeled with P^32^-gamma-ATP by T4 polynucleotide kinase (NEB, Ipswich, MA, USA). A miRNA Marker Probe (21-nucleotide; NEB, Ipswich, MA, USA) was used for sRNA size determination. The prepared membrane filters were hybridized at 42°C overnight, then washed twice at 55°C with washing buffer containing 2× SSC and 2% SDS. Membranes were then exposed to phosphorscreens and scanned with a Typhoon TRIO Variable Mode Imager (GE Healthcare). Membrane exposure time was adjusted, dependent on signal intensity.

### Degradome analysis

For degradome sequencing, mixed RNAs with equal amounts from leaf, root, flower and fruit tissues were used. After adaptor-trimming and genomic mapping as done for the sRNA data, the Cleaveland pipeline 2.0 [55] was optimized to analyze the degradome sequencing data in collaboration with Targetfinder 1.6 [86]. The alignment score threshold was set to 4.5 for conserved and less conserved miRNAs (except for two *ARF *targets of miR167 and two *MYB *targets of miR858, for which the score was 5) and to 5 for novel and candidate miRNAs. The apple consensus gene set and the annotation information of miRNA target genes were retrieved from Genome Database for Rosaceae (GDR). Degradome data were normalized to transcripts per billon (TPB).

### RLM-5'-RACE

Following the manufacturer's instructions for the FirstChoice RLM-RACE Kit (Ambion, Austin, TX, USA), 2 μg of total RNA isolated from apple flower was used for ligating 5' RNA adaptors at 15°C overnight. Two specific primers (Table S9 in Additional file 1) were designed to conduct nested PCRs, and PCR products were cloned to the pGEM-easy vector (Promega, Madison, WI, USA) and sequenced by Bechman Coulter Genomics (Danvers, MA, USA).

## Abbreviations

ARF: auxin response factor; bHLH: basic helix-loop-helix; DCL: Dicer-like protein; miRNA: microRNA; PCR: polymerase chain reaction; RDR: RNA-dependent RNA polymerase; RISC: RNA-induced silencing complex; RLM-5'-RACE: RNA ligation-mediated 5' rapid amplification of cDNA ends; RPM: reads per million genome-matched reads; siRNA: small interfering RNA; sRNA: small RNA; tasiRNA: trans-acting small interfering RNA.

## Competing interests

The authors declare that they have no competing interests.

## Authors' contributions

RX and ZL initiated the research. RX, ZL, EB and YA designed the experiments. RX performed the computational analyses. HZ, RX and ZL carried out the biological experiments. RX, EB and ZL interpreted the results and prepared the manuscript. All authors have read and approved the manuscript for publication.

## Supplementary Material

Additional file 1 **Supplemental Tables S1 to S9**. Table S1: reads statistics in four libraries. Table S2: read length distribution in each conserved miRNA family. Table S3: homologous sequences for known miRNAs. Table S4: known miRNAs with good stem-loop structure predicted. Table S5: detailed list of novel and candidate miRNAs found in apple. Table S6: targets of apple miRNAs (or families; detailed list). Table S7: *MYB *genes targeted by miR828, mi858, and miR159. Table S8: targets of *MYB*-derived phased siRNAs. Table S9: RNA gel blotting probes and RLM-5'-RACE primers. Click here for file

Additional file 2 **Supplemental Figures S1 to S5**. Figure S1: diversity and size distribution of redundant and unique sRNAs. Figure S2: distribution of 21-nucleotide phasing siRNAs along apple *TAS *genes. Figure S3: multiple alignment of R3 repeat domain for 251 apple and 129 *Arabidopsis *MYBs. Figure S4: T-plots for targets of miR828, miR858 and tasiARF. Figure S5: distribution analysis of siRNAs derived from the ten miR828-targeted *MYB *genes. Click here for file

Additional file 3 **Predicted secondary structures of the apple-specific miRNAs**. This file contains all the secondary stem-loop structures for the apple-specific miRNAs. The miRNA and miRNA* sequences are denoted in red and green, respectively. Click here for file

Additional file 4 **Mapping plots of novel apple miRNAs**. This file contains all the mapping plots illustrating the read distribution along the precursor region of novel apple miRNAs. Click here for file
