# Supplementary material for: Apple miRNAs and tasiRNAs with novel regulatory networks
Source: Genome Biol. 2012 Jun 15;13(6):R47. doi: 10.1186/gb-2012-13-6-r47 (PMC3446319; doi:10.1186/gb-2012-13-6-r47)
Supplement: Additional file 2 — Supplemental Figures S1 to S5. Figure S1: diversity and size distribution of redundant and unique sRNAs. Figure S2: distribution of 21-nucleotide phasing siRNAs along apple TAS genes. Figure S3: multiple alignment of R3 repeat domain for 251 apple and 129 Arabidopsis MYBs. Figure S4: T-plots for targets of miR828, miR858 and tasiARF. Figure S5: distribution analysis of siRNAs derived from the ten miR828-targeted MYB genes. [file gb-2012-13-6-r47-S2.PDF]

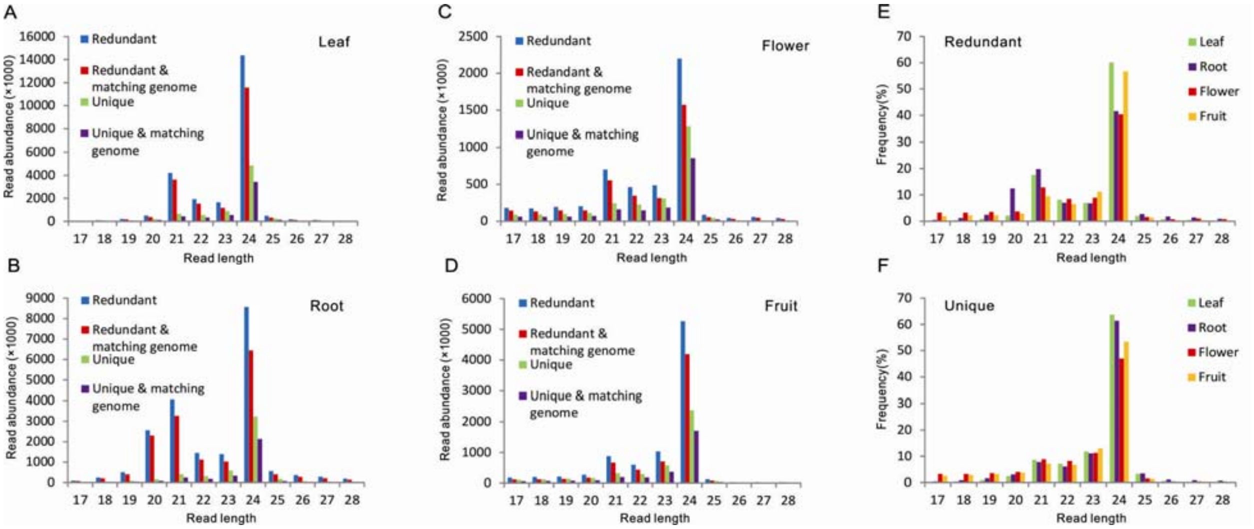

Figure S1. Diversity and size distribution of redundant and unique sRNA reads.

(A-D) Read abundances of redundant and unique sRNAs from leaf, root, flower and fruit libraries are shown in panels. (**E-F)** The comparison of read size distribution among libraries as expressed as a percentage of the total number of redundant or unique read sequences for each tissue.

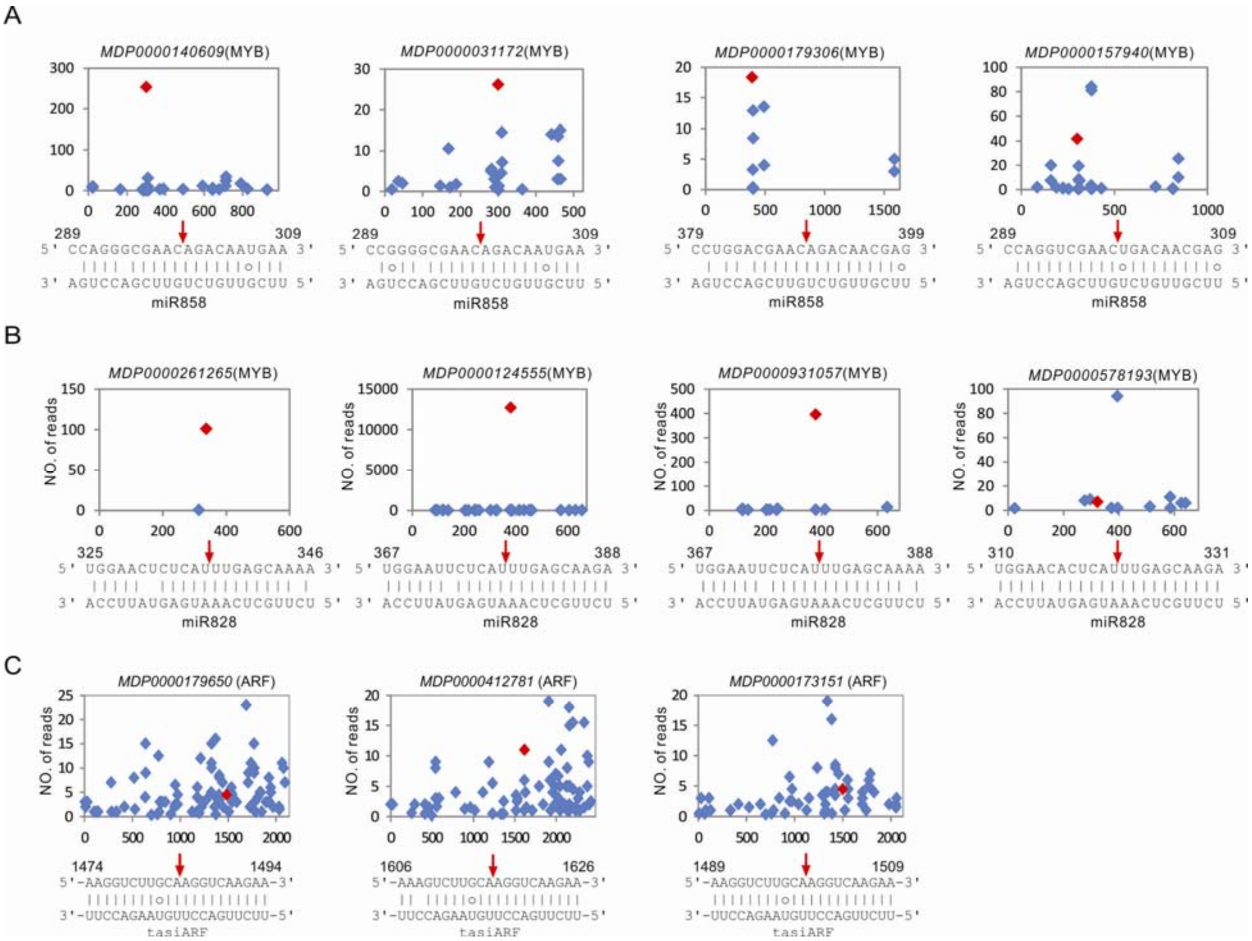

Figure S2 T-plots for targets of miR858, miR828 and tasiARF

Paring between miRNA (siRNA) and complementary target site is displayed below the T-plots. The position of cleavage sites is indicated by the red diamond in the plot and the red arrow in the pairing between target site and miRNA. **A.** Targets of miR858. **B.** Targets of miR828. **C.** Targets of tasiARF.

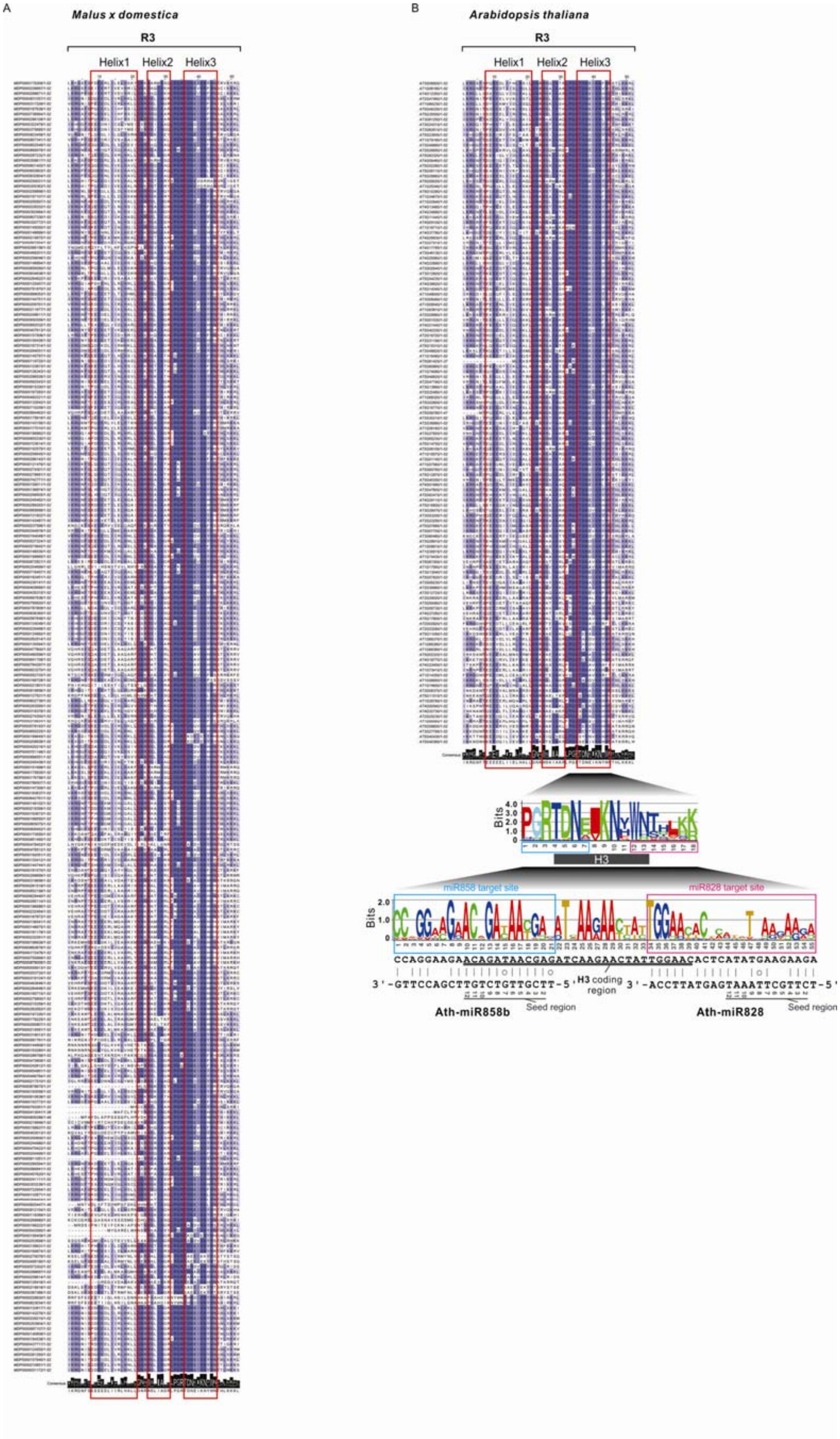
A
Malus x domestica
R3
Helix1
Helix2
Helix3
B
Arabidopsis thaliana
R3
Helix1
Helix2
Helix3
Bits
H3
miR858 target site
miR828 target site
Bits
CCAGGAAGAACAGATAACGAGATCAAGAACTATTGGAACACTCATATGAAGAGA
3'-GTTCCAGCTTGTCTGTTGCTT-5'
H3 coding region
3'-ACCTTATGAGTAAATTCGTTCT-5'
Seed region
Ath-miR858b
Seed region
Ath-miR828

Figure S3 Multiple alignment of the R3 repeat domain for 251 apple and 129 *Arabidopsis* MYBs

A. Multiple alignment of R3 repeat domain for 251 apple MYBs; B. Multiple alignment of R3 repeat domain for 129 *Arabidopsis* MYBs and sequence logos of the miRNA co-targeting region. The position of three helixes in the R3 repeat domain is indicated in red boxes. The paring between the consensus sequence and the miRNA is illustrated. The third helix (H3) region, miRNA target sites and their seed sequences are marked.

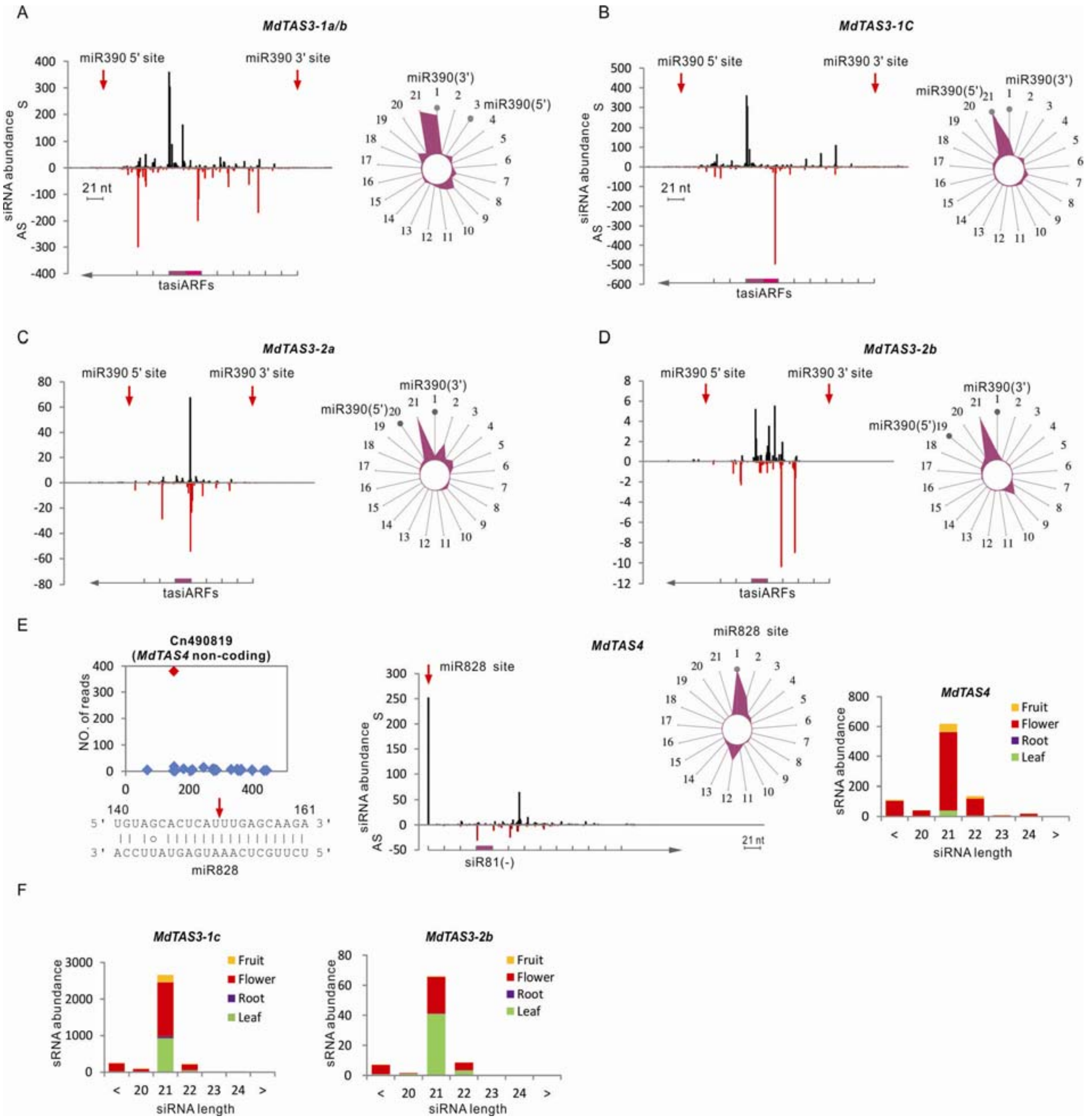

Figure S4 Distribution of 21-nt phasing siRNAs along apple TAS genes.

A-D. The number of 21-nt sRNA reads with 5' residues at each position along a *TAS3* locus was plotted for the sense (S) and antisense (AS) strands. Radar plot displays the abundance of reads corresponding to each of the 21 possible phasing registers, with the 5' end of the miR390-guided cleavage of the 3' complementary site defined as register 1. The total number of small RNAs mapping to that register is plotted as relative distance from the center. The registers proceed clockwise from 5' to 3'. The 5' complementary site of miR390 and the position of two conserved tasiARFs are also noted. **E.** T-plot for the miR828-guided cleavage of *MdTAS4* is shown on left with paring between miR828 and complementary target site displayed below. The position of cleavage site was shown by the red diamond

in the plot and the red arrow in the paring between target site and miRNA. The number of 21-nt sRNA reads with 5' residues at each position along the *TAS4* locus was plotted for the sense (S) and antisense (AS) strands in the middle. Radar plot displays the abundance of reads corresponding to each of the 21 possible phasing registers, with the cleavage site of miR828 defined as register 1. The length distribution of siRNAs matched to the *TAS4* locus is illustrated at right. **F.** The length distribution of siRNAs matched to *MdTAS3-1c* and *MdTAS3-2c*.

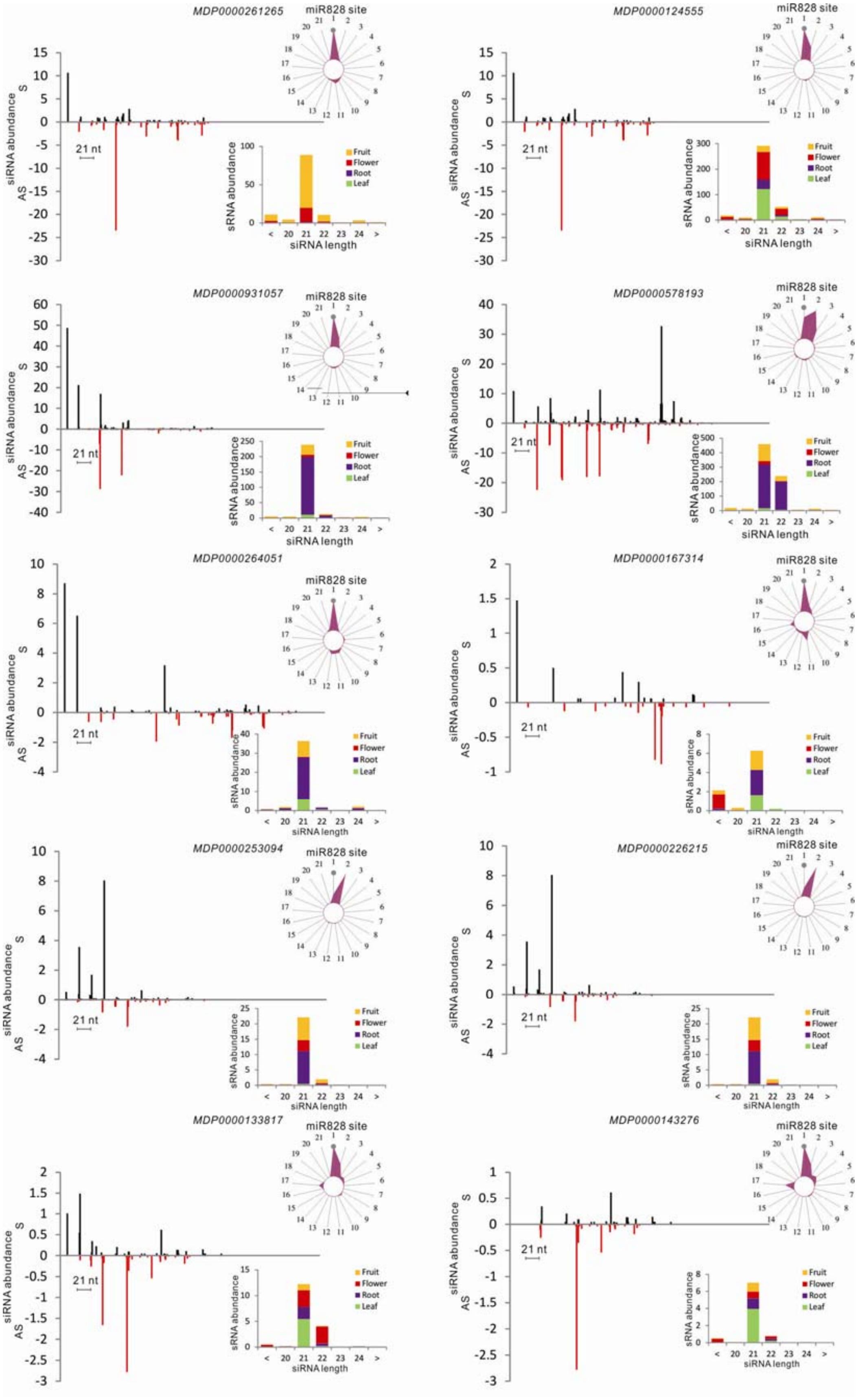

MDP0000261265
MDP0000124555
MDP0000931057
MDP0000578193
MDP0000264051
MDP0000167314
MDP0000253094
MDP0000226215
MDP0000133817
MDP0000143276
miR828 site
siRNA abundance
S
AS
21 nt
sRNA abundance
siRNA length
Fruit
Flower
Root
Leaf
<
20
21
22
23
24
>

Figure S5 Read distribution analysis of siRNAs derived from miR828-targeted *MYB* genes.

The number of 21-nt sRNA reads with 5’ residues at each position along a certain *MYB* gene is plotted for the sense (S) and antisense (AS) strands. Radar plot displayed the abundance of reads corresponding to each of the 21 possible phasing registers, with the cleavage site of miR828 defined as register 1. The length distribution of siRNAs matched to each *MYB* genes is illustrated in the inset bar graph for each *MYB*.
