# Supplementary material for: Apple miRNAs and tasiRNAs with novel regulatory networks
Source: Genome Biol. 2012 Jun 15;13(6):R47. doi: 10.1186/gb-2012-13-6-r47 (PMC3446319; doi:10.1186/gb-2012-13-6-r47)

Name: Mdo_miRC1

Contig: MDC006505.260

Position: 3797

Abundance: 24349

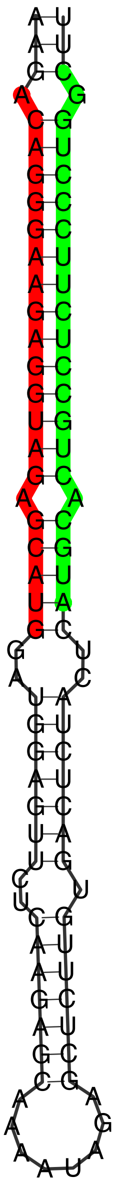

Name: miRC1

Contig: MDC006859.349

Position: 17398

Abundance: 24349

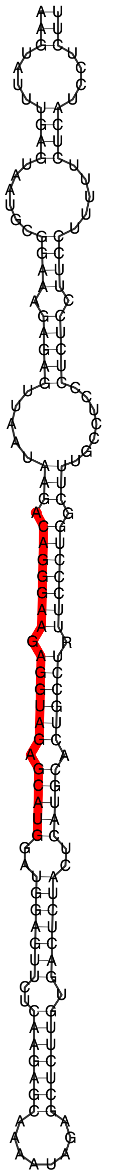

Name: Mdo_miRC1

Contig: MDC016595.160

Position: 1339

Abundance: 24349

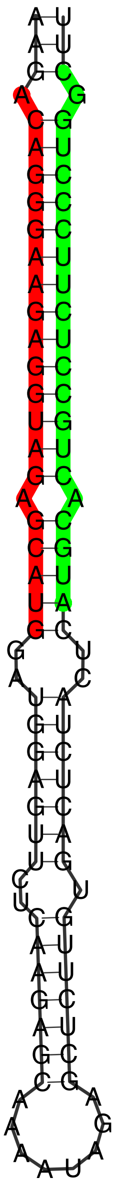

Name: Mdo_miRC2

Contig: MDC018599.370

Position: 9369

Abundance: 8341

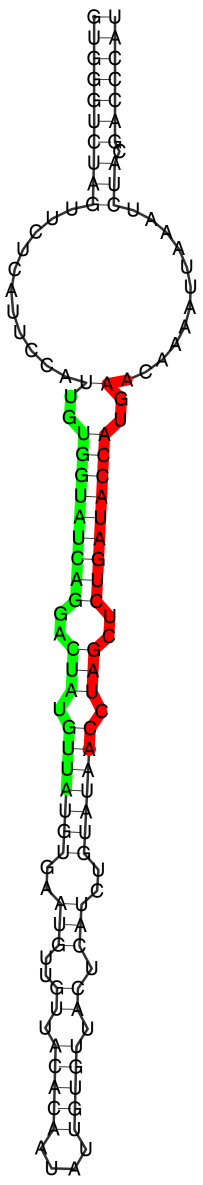

Name: miRC3

Contig: MDC017130.228

Position: 6809

Abundance: 2751

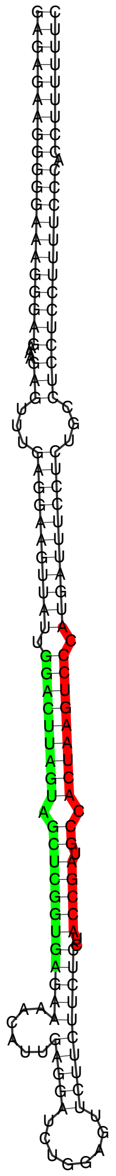

Name: miRC3

Contig: MDC019300.125

Position: 1308

Abundance: 2751

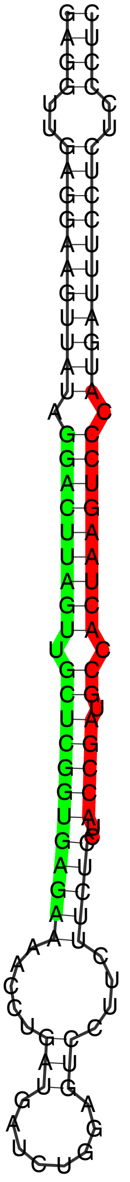

Name: miRC3

Contig: MDC019300.66

Position: 1894

Abundance: 2751

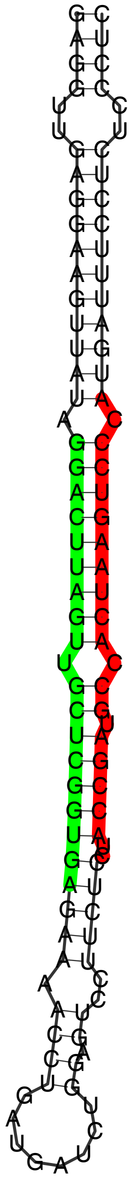

Name: miRC4

Contig: MDC019554.272

Position: 10154

Abundance: 1543

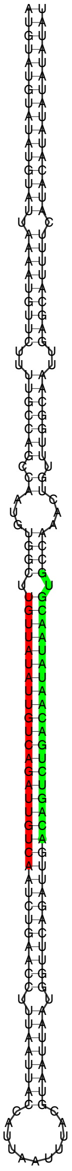

Name: miRC4

Contig: MDC017840.207

Position: 3677

Abundance: 1537

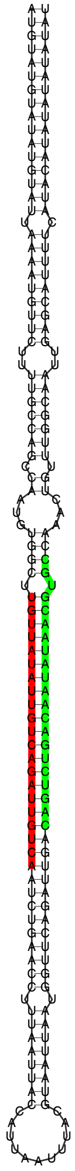

Name: miRC5

Contig: MDC006350.123

Position: 2997

Abundance: 1490

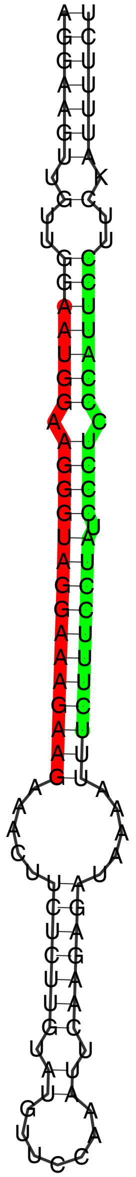

Name: miRC6a

Contig: MDC009272.709

Position: 4184

Abundance: 1294

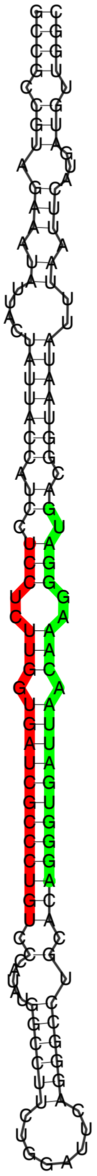

Name: miRC6a

Contig: MDC009272.711

Position: 1281

Abundance: 1294

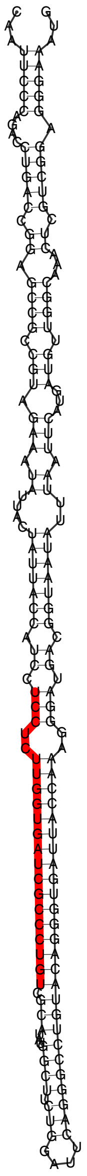

Name: miRC6a

Contig: MDC025804.16

Position: 2961

Abundance: 1294

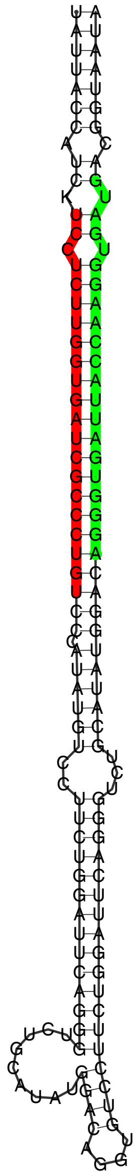

Name: miRC6b

Contig: MDC006081.961

Position: 466

Abundance: 385

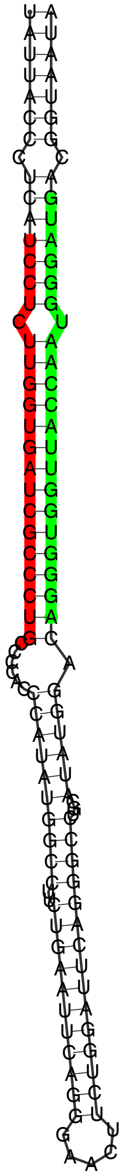

Name: miRC6b

Contig: MDC006416.180

Position: 817

Abundance: 385

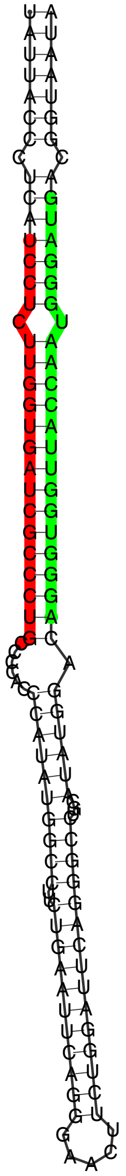

Name: miRC6b

Contig: MDC009272.295

Position: 602

Abundance: 385

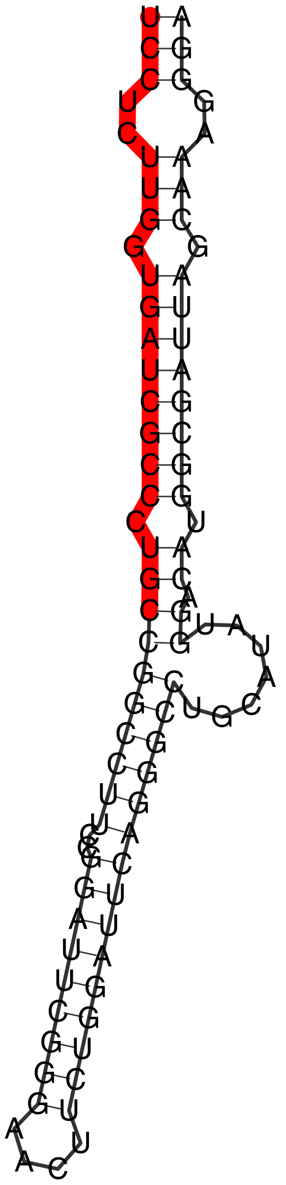

Name: miRC6b

Contig: MDC009272.679

Position: 846

Abundance: 385

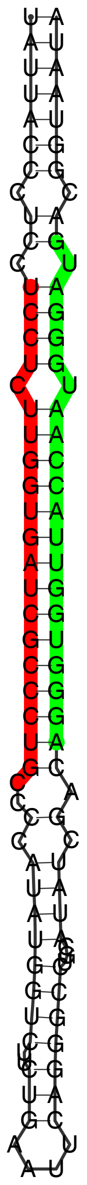

Name: miRC6b

Contig: MDC027090.15

Position: 1078

Abundance: 385

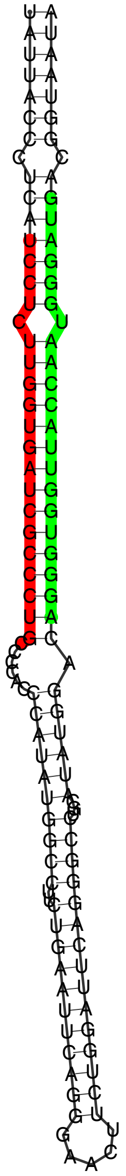

Name: miRC7

Contig: MDC009778.59

Position: 4735

Abundance: 1270

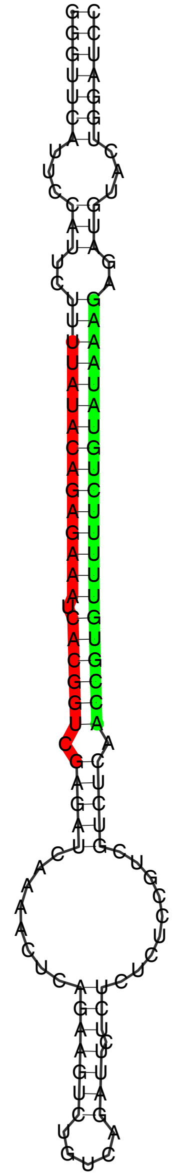

Name: miRC7

Contig: MDC022484.54

Position: 221

Abundance: 1270

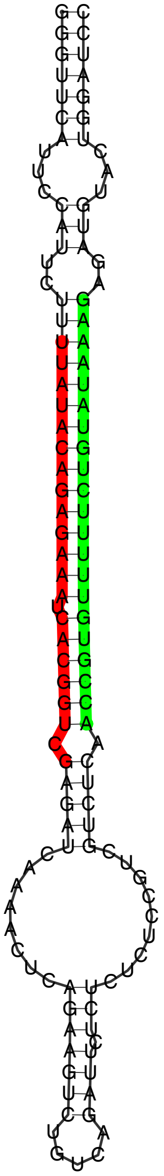

Name: miRC8

Contig: MDC001018.301

Position: 2490

Abundance: 248

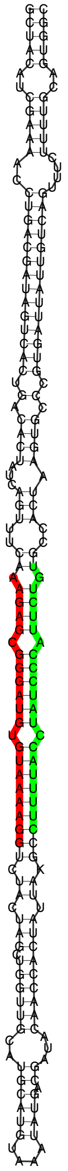

Name: miRC8

Contig: MDC010449.276

Position: 9302

Abundance: 248

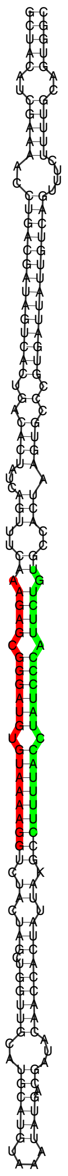

Name: miRC9

Contig: MDC011178.406

Position: 16699

Abundance: 231

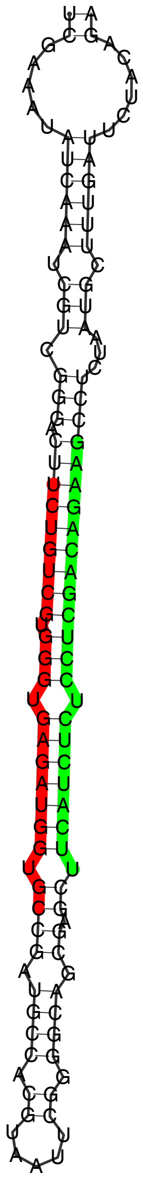

Name: miRC10

Contig: MDC026449.10

Position: 340

Abundance: 173

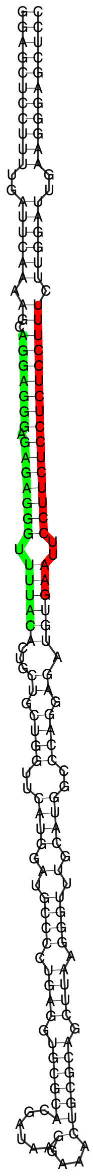

Name: miRC11

Contig: MDC005581.168

Position: 2657

Abundance: 100

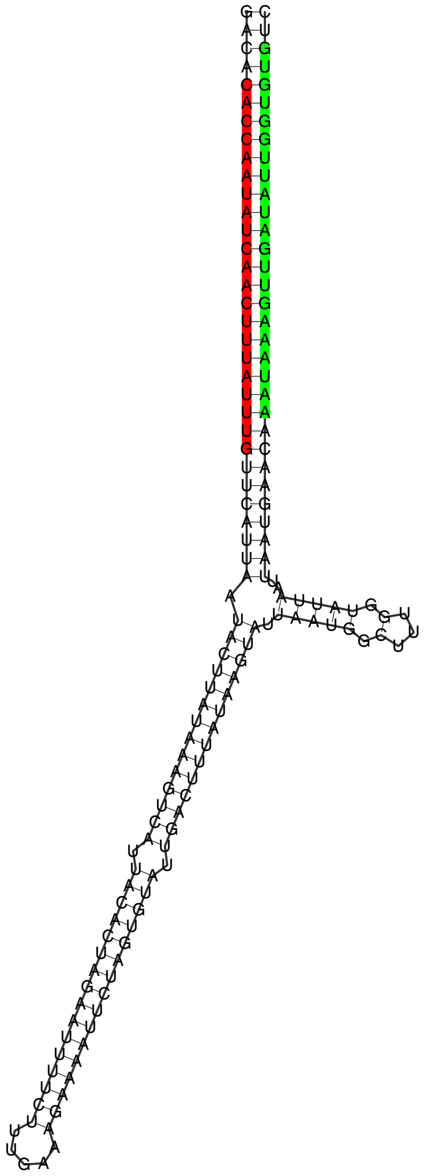

Name: miRC11

Contig: MDC001716.95

Position: 7475

Abundance: 100

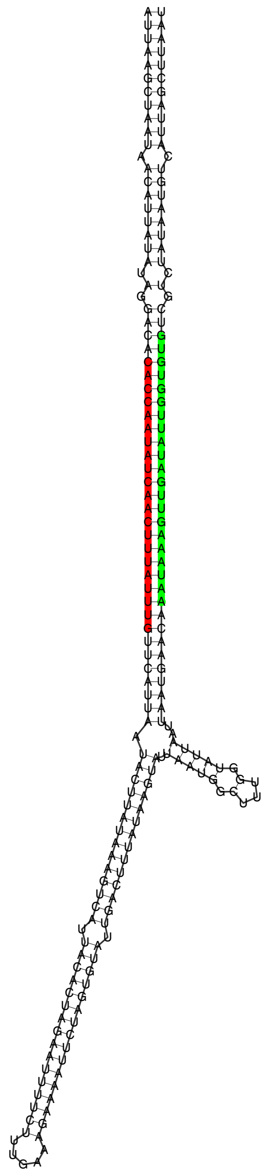

Name: miRC12

Contig: MDC003092.251

Position: 8878

Abundance: 73

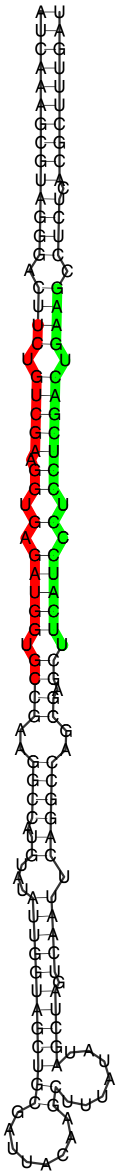

Name: miRC12

Contig: MDC003092.253

Position: 6600

Abundance: 73

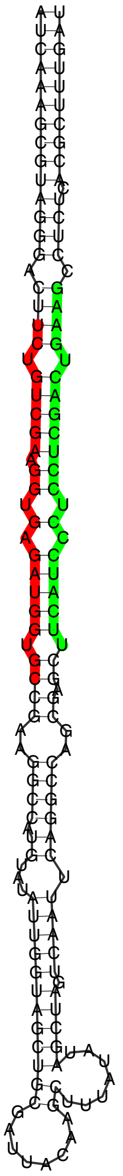

Name: Mdo_miRC13

Contig: MDC009318.175

Position: 3741

Abundance: 60

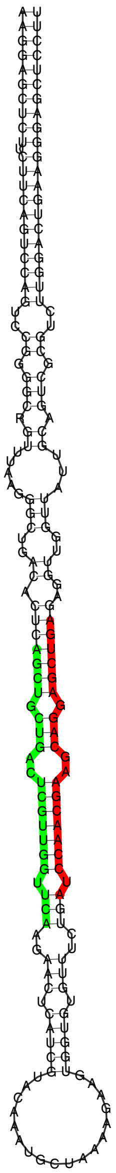

Name: miRC14

Contig: MDC008558.180

Position: 6507

Abundance: 38

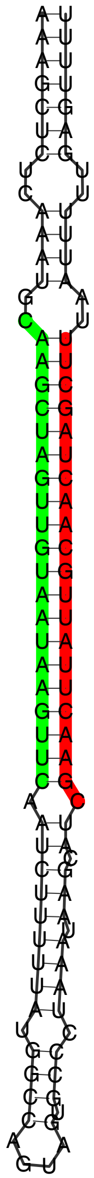

Name: miRC15

Contig: MDC014075.204

Position: 26060

Abundance: 27

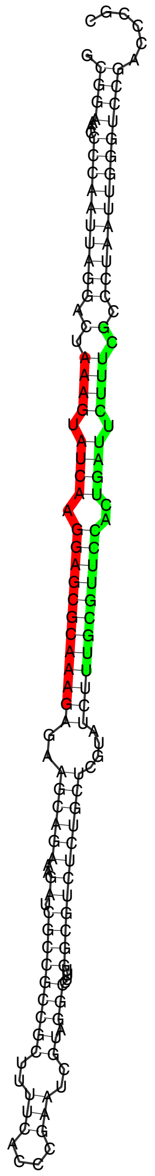

Name: miRC16

Contig: MDC009589.307

Position: 1975

Abundance: 22

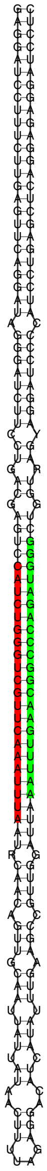

Name: miRC16

Contig: MDC016571.174

Position: 3713

Abundance: 22

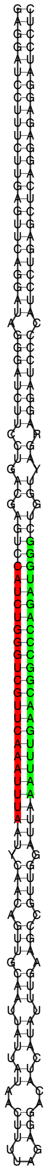

Name: miRC16

Contig: MDC025620.10

Position: 649

Abundance: 22

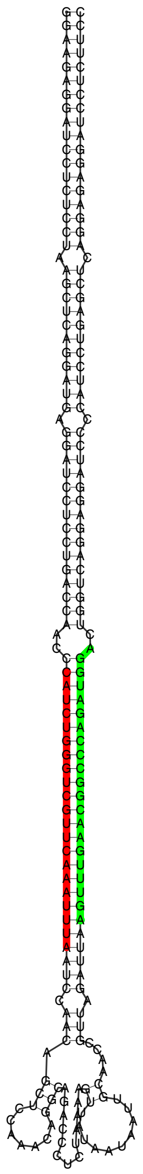

Name: miRC17

Contig: MDC005391.194

Position: 11429

Abundance: 11

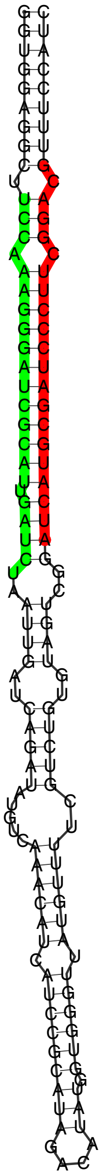

Name: miRC17

Contig: MDC005391.195

Position: 349

Abundance: 11

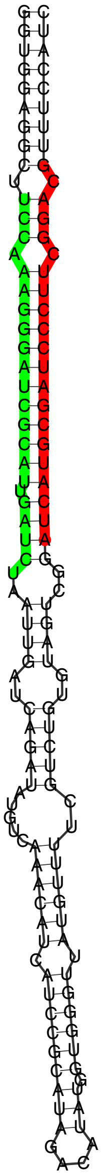

Name: miRC17

Contig: MDC019461.80

Position: 15051

Abundance: 11

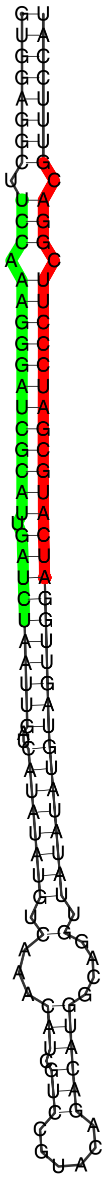

Name: miRC18

Contig: MDC012422.128

Position: 849

Abundance: 8

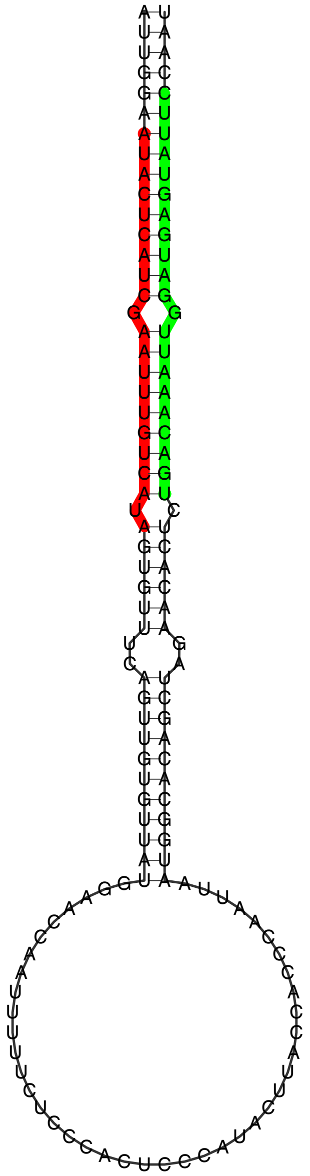

Name: miRC18

Contig: MDC021812.77

Position: 16858

Abundance: 8

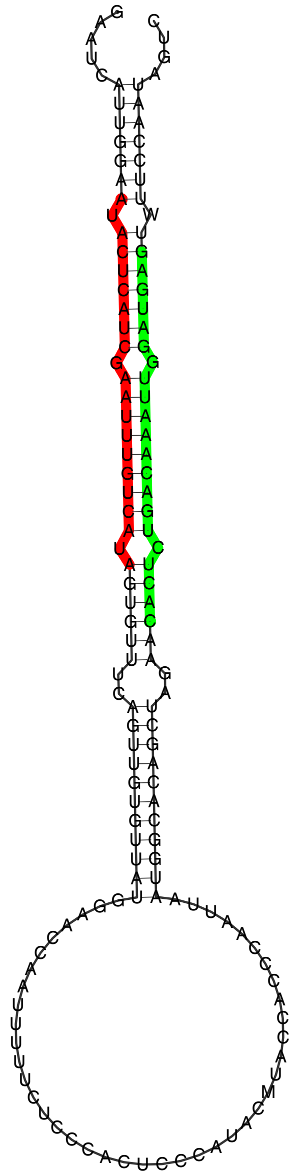

Name: miRC19

Contig: MDC016463.170

Position: 5187

Abundance: 7

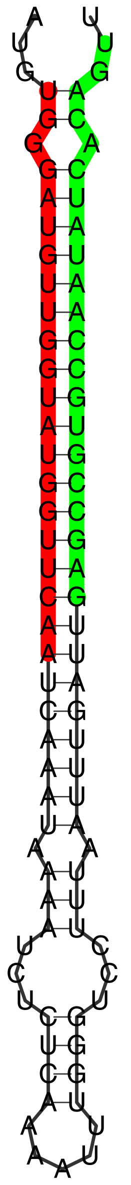

Name: miRC20

Contig: MDC001494.456

Position: 33010

Abundance: 6

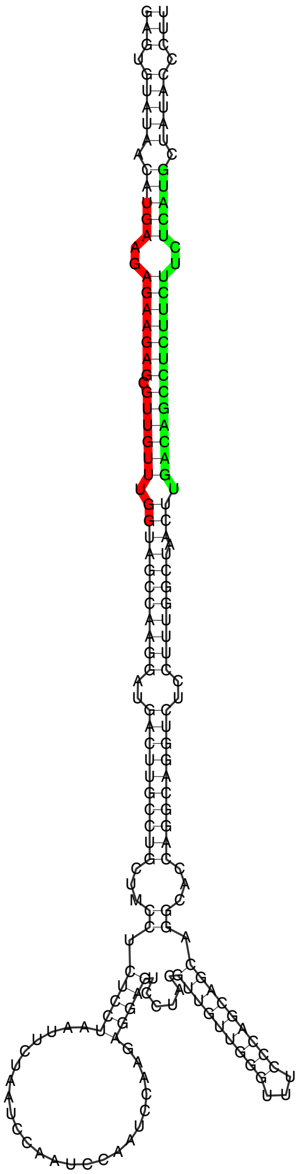

Name: miRC20

Contig: MDC013464.188

Position: 2258

Abundance: 6

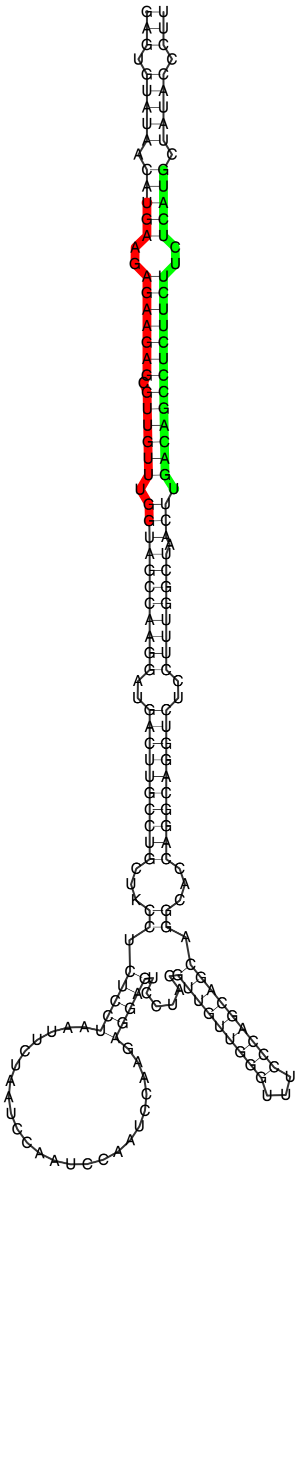

Name: miRC21

Contig: MDC006081.432

Position: 219

Abundance: 2

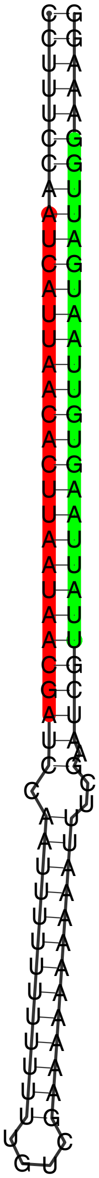

Name: miRC22

Contig: MDC016302.308

Position: 5368

Abundance: 80

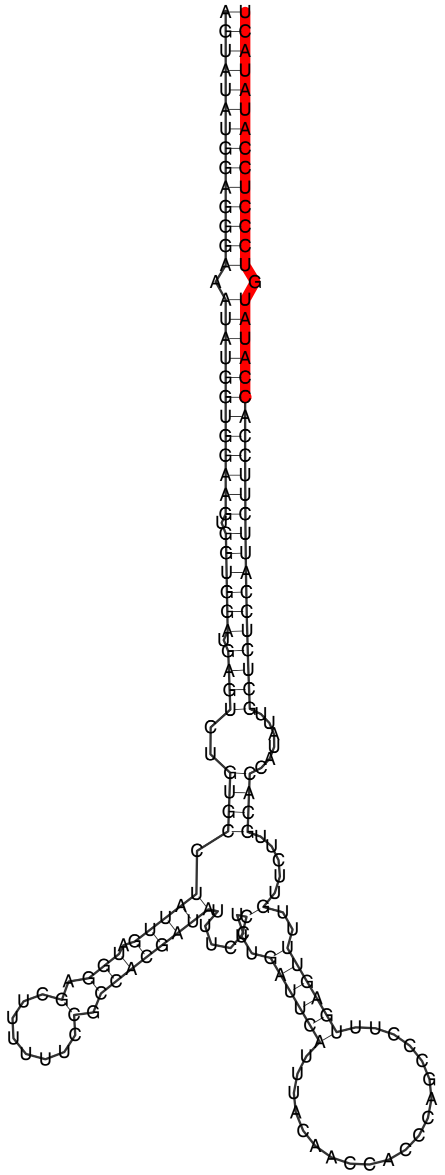

Name: miRC23

Contig: MDC020884.221

Position: 4086

Abundance: 48

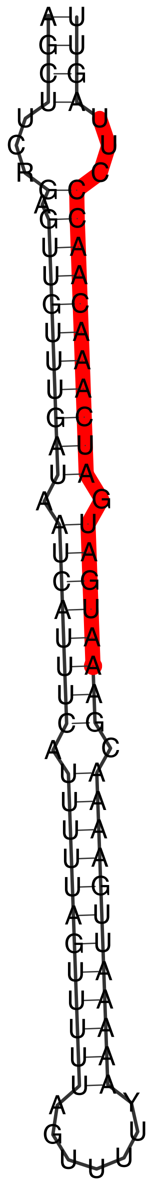

Name: miRC24

Contig: MDC001394.253

Position: 378

Abundance: 40

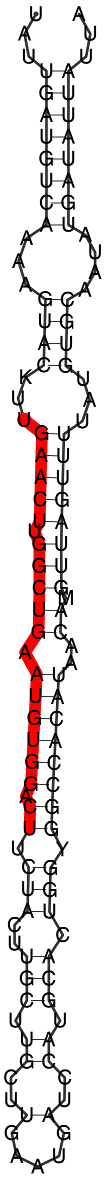

Name: miRC24

Contig: MDC001394.350

Position: 4731

Abundance: 40

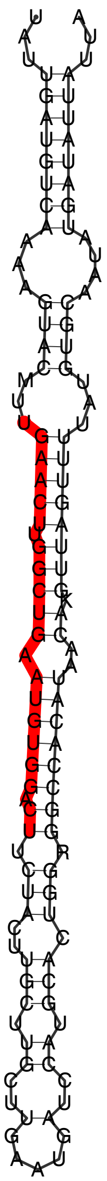

Name: miRC25

Contig: MDC017130.228

Position: 9764

Abundance: 24

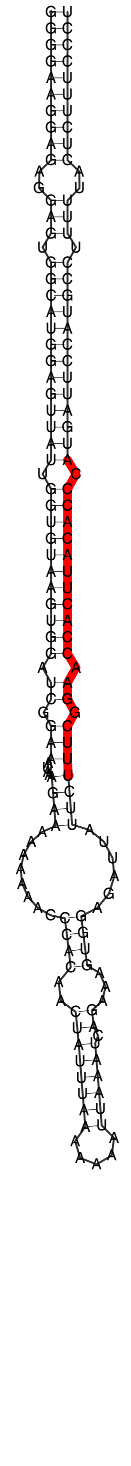

Name: miRC26

Contig: MDC017130.228

Position: 9967

Abundance: 15

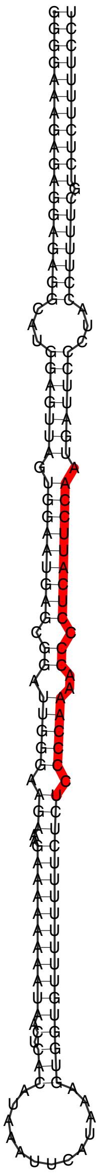

Name: miRC27

Contig: MDC019485.283

Position: 7997

Abundance: 12

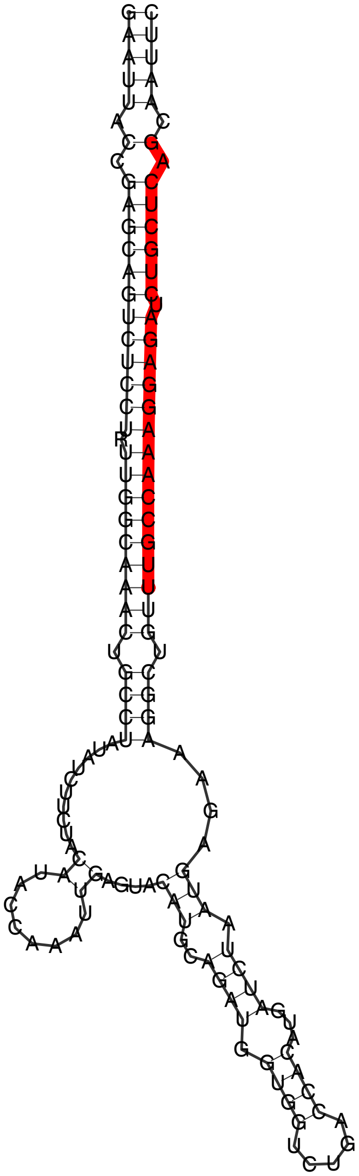

Name: miRC28

Contig: MDC007946.169

Position: 672

Abundance: 11

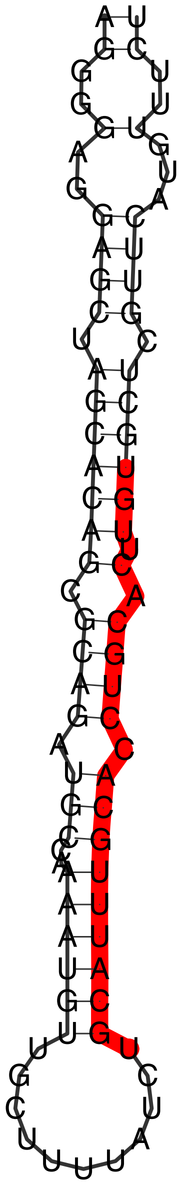

Name: miRC30

Contig: MDC004268.215

Position: 9006

Abundance: 10

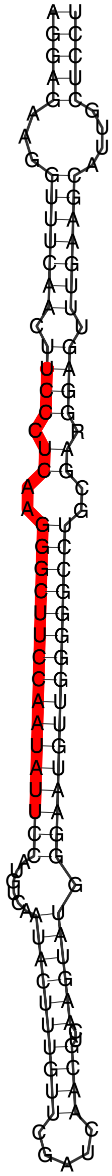

Name: miRC31

Contig: MDC005072.383

Position: 9885

Abundance: 8

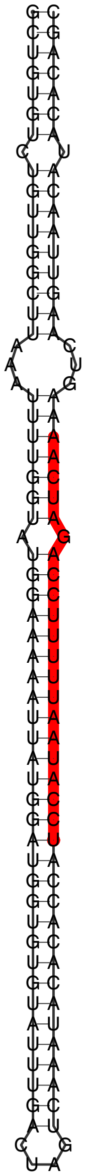

Name: miRC32

Contig: MDC006935.286

Position: 27531

Abundance: 7

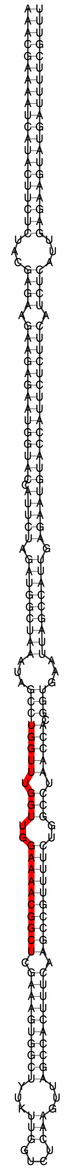

Name: miRC32

Contig: MDC010077.377

Position: 4558

Abundance: 7

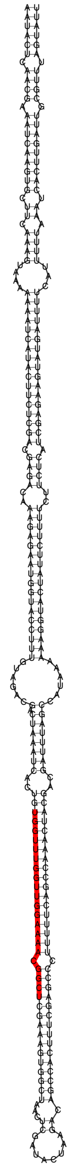

Name: miRC32

Contig: MDC015760.248

Position: 5247

Abundance: 7

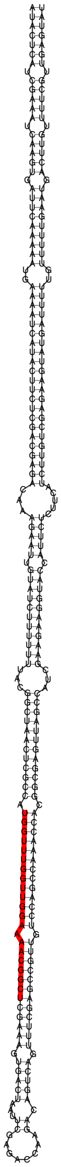

Name: miRC32

Contig: MDC015809.231

Position: 4394

Abundance: 7

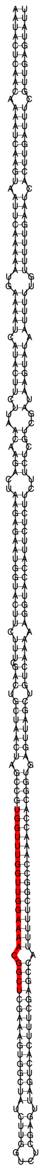

Name: miRC33

Contig: MDC009589.310

Position: 3312

Abundance: 6

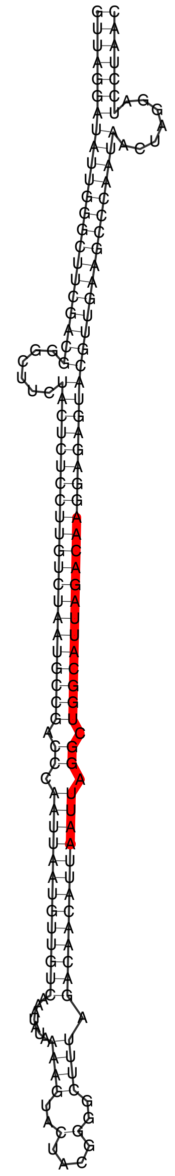

Name: miRC33

Contig: MDC009589.320

Position: 6342

Abundance: 6

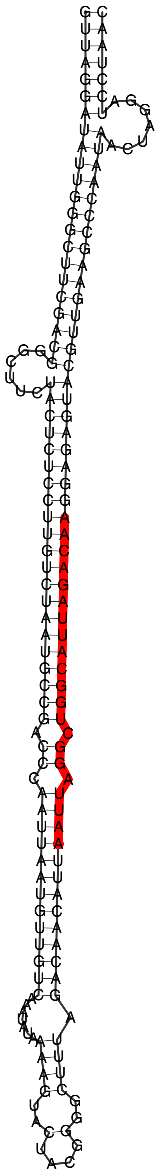

Name: miRC34

Contig: MDC010150.221

Position: 29453

Abundance: 6

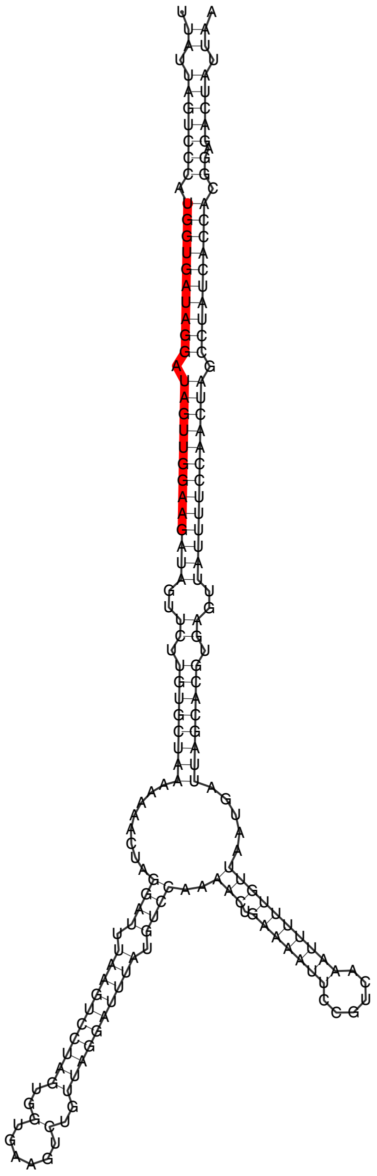

Name: miRC35

Contig: MDC001086.52

Position: 12332

Abundance: 5

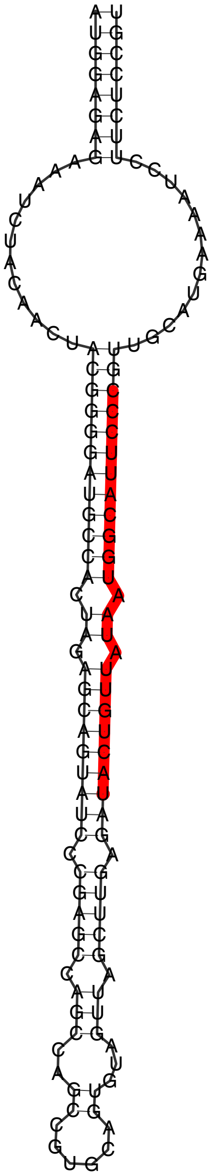

Name: miRC36

Contig: MDC015454.116

Position: 3773

Abundance: 5

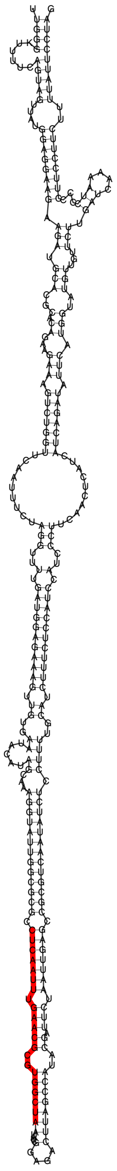

Name: miRC37

Contig: MDC000614.265

Position: 5670

Abundance: 4

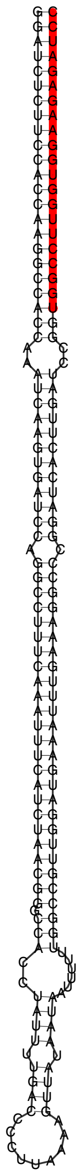

Name: miRC37

Contig: MDC011433.387

Position: 9452

Abundance: 4

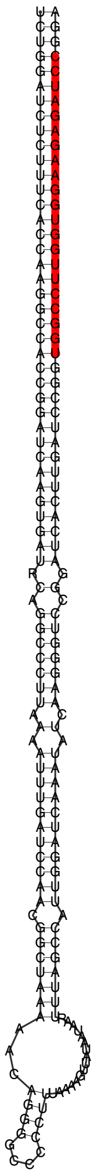

Name: miRC37

Contig: MDC012039.280

Position: 36010

Abundance: 4

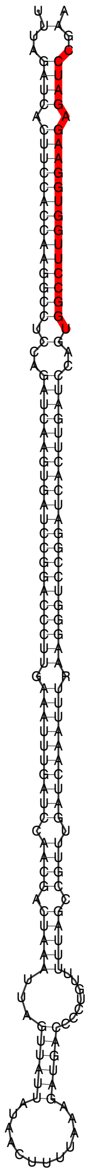

Name: miRC37

Contig: MDC013874.469

Position: 13657

Abundance: 4

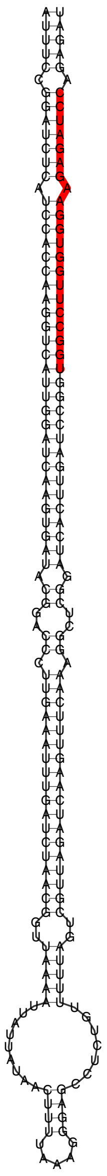

Name: miRC37
Contig: MDC015646.206
Position: 32405
Abundance: 4

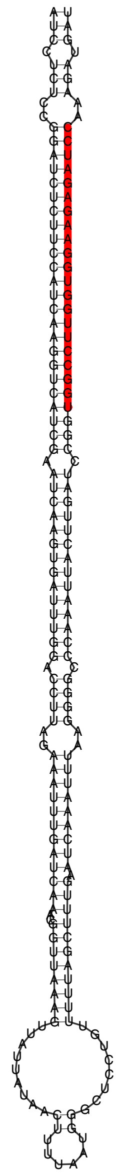

Name: miRC37

Contig: MDC016472.111

Position: 4859

Abundance: 4

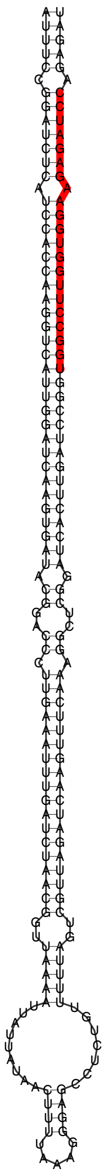

Name: miRC38

Contig: MDC018501.179

Position: 2535

Abundance: 4

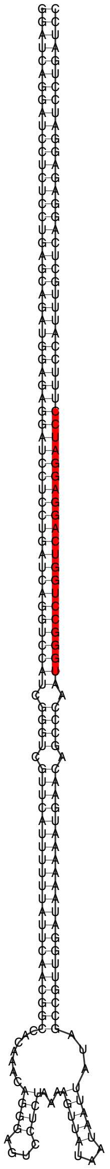

Name: miRC38

Contig: MDC018580.433

Position: 345

Abundance: 4

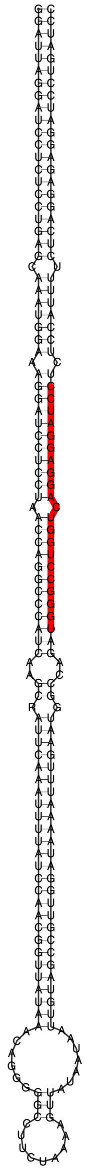

Name: miRC39

Contig: MDC018873.313

Position: 10447

Abundance: 10

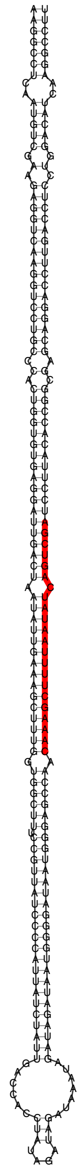

Name: miRC39

Contig: MDC011810.169

Position: 42284

Abundance: 3

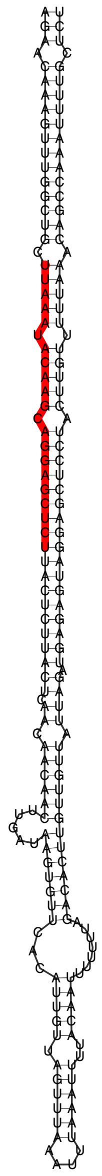

Name: miRC40

Contig: MDC009540.166

Position: 1015

Abundance: 3

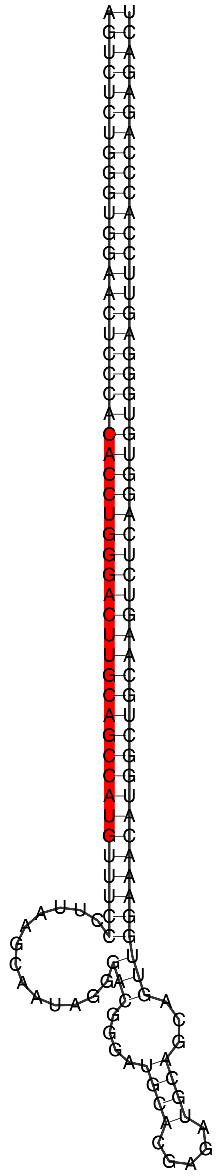

Name: miRC41

Contig: MDC009746.72

Position: 18725

Abundance: 3

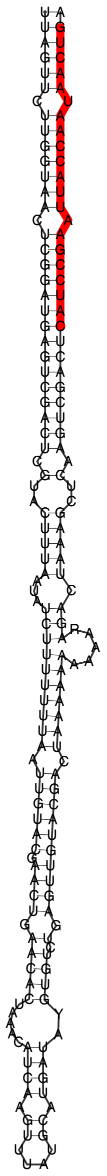

Name: miRC42

Contig: MDC013676.252

Position: 6707

Abundance: 3

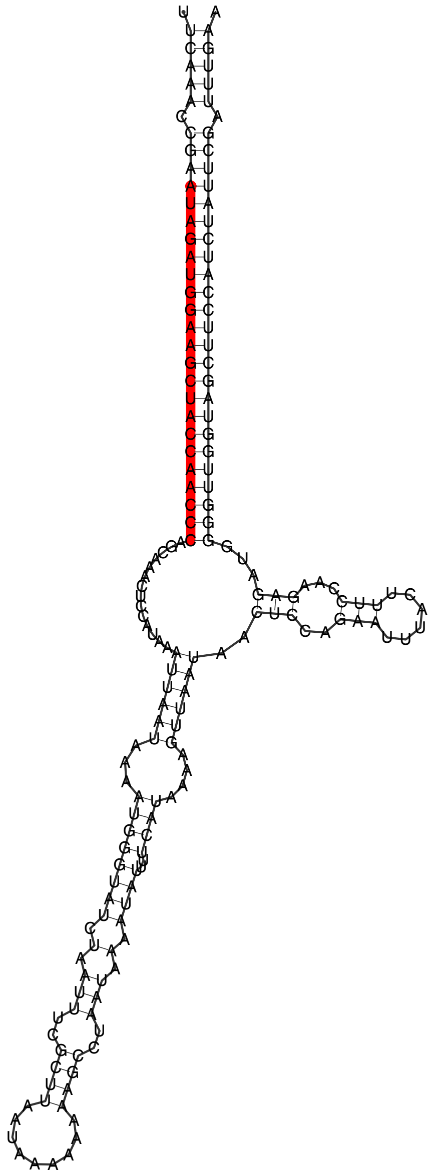

Supplement: Additional file 3 — Predicted secondary structures of the apple-specific miRNAs. This file contains all the secondary stem-loop structures for the apple-specific miRNAs. The miRNA and miRNA* sequences are denoted in red and green, respectively. [file gb-2012-13-6-r47-S3.PDF]
