# Supplementary material for: Apple miRNAs and tasiRNAs with novel regulatory networks
Source: Genome Biol. 2012 Jun 15;13(6):R47. doi: 10.1186/gb-2012-13-6-r47 (PMC3446319; doi:10.1186/gb-2012-13-6-r47)

Mapping plots for novel miRNAs

Mapping results for a precursor sequence (-150 ~ 150 bp) were plotted. The x-axis is the position (0-320/321 bp) and the y-axis is the count of reads matched to a specific position. The 150-170/171 bp is the position for miRNA mature sequence, and miRNA* position is marked in a blue box.

miRC1

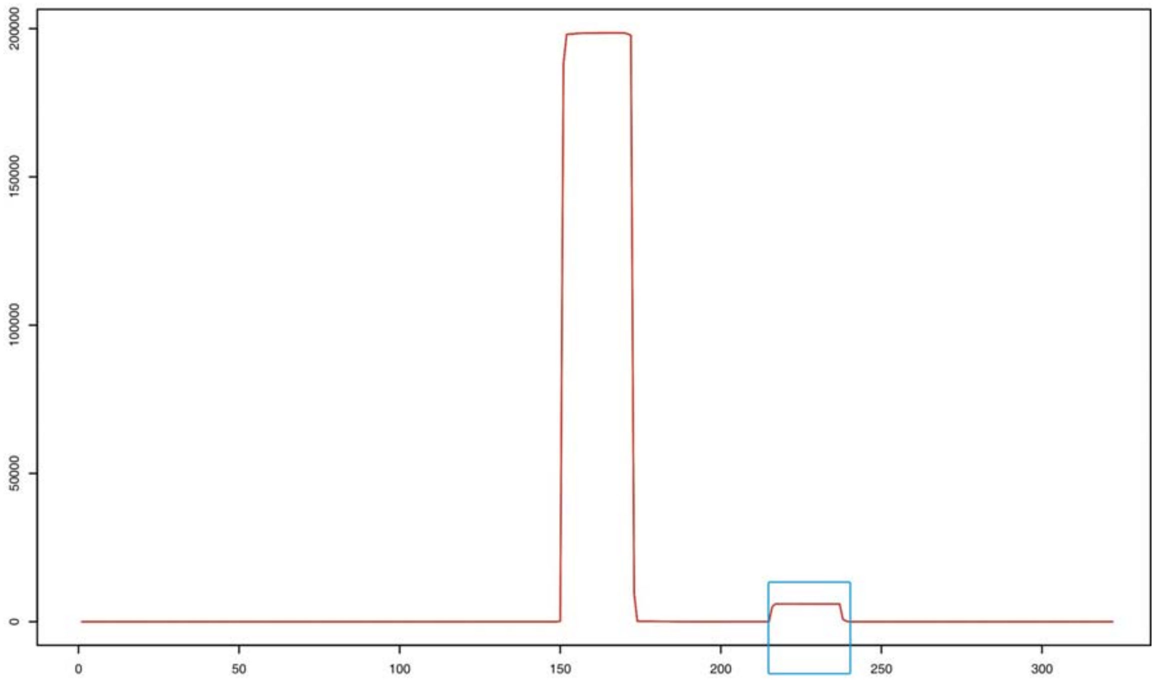

miRC2

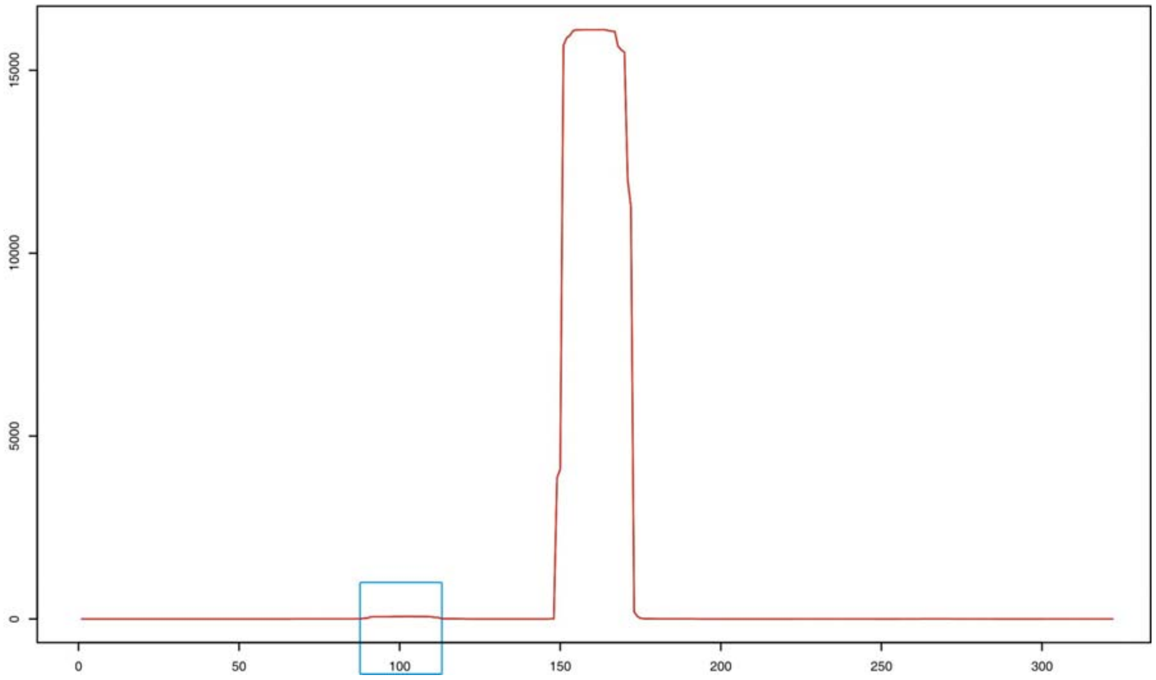

miRC3

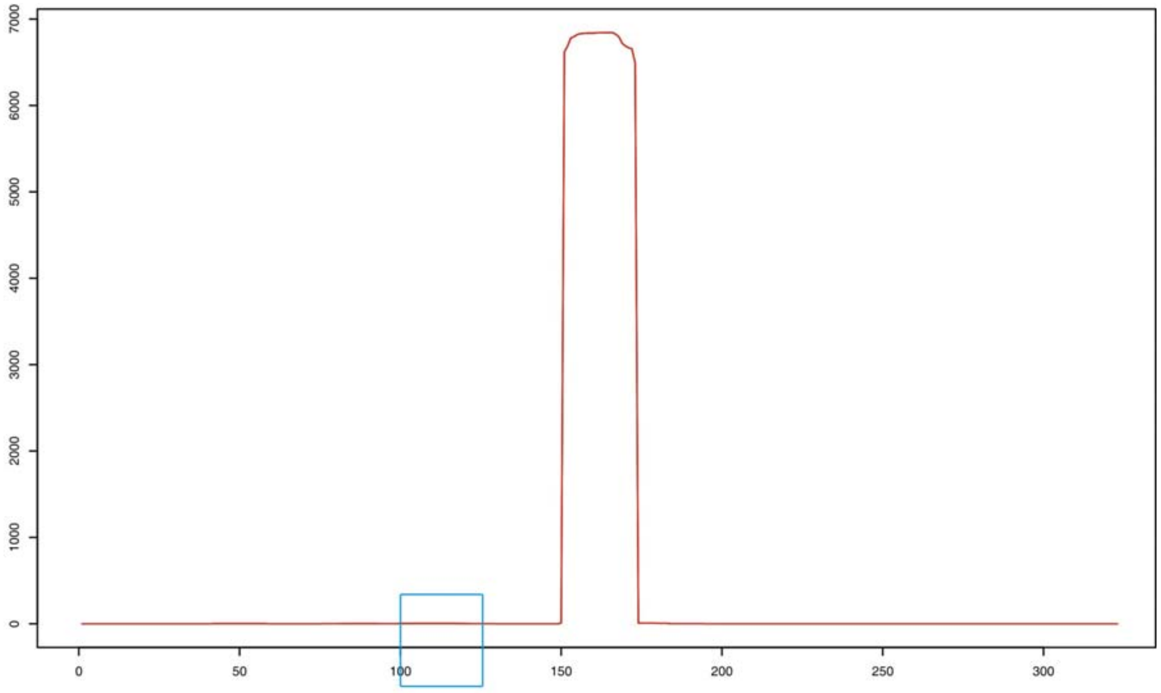

7000
6000
5000
4000
3000
2000
1000
0
0
50
100
150
200
250
300

miRC4

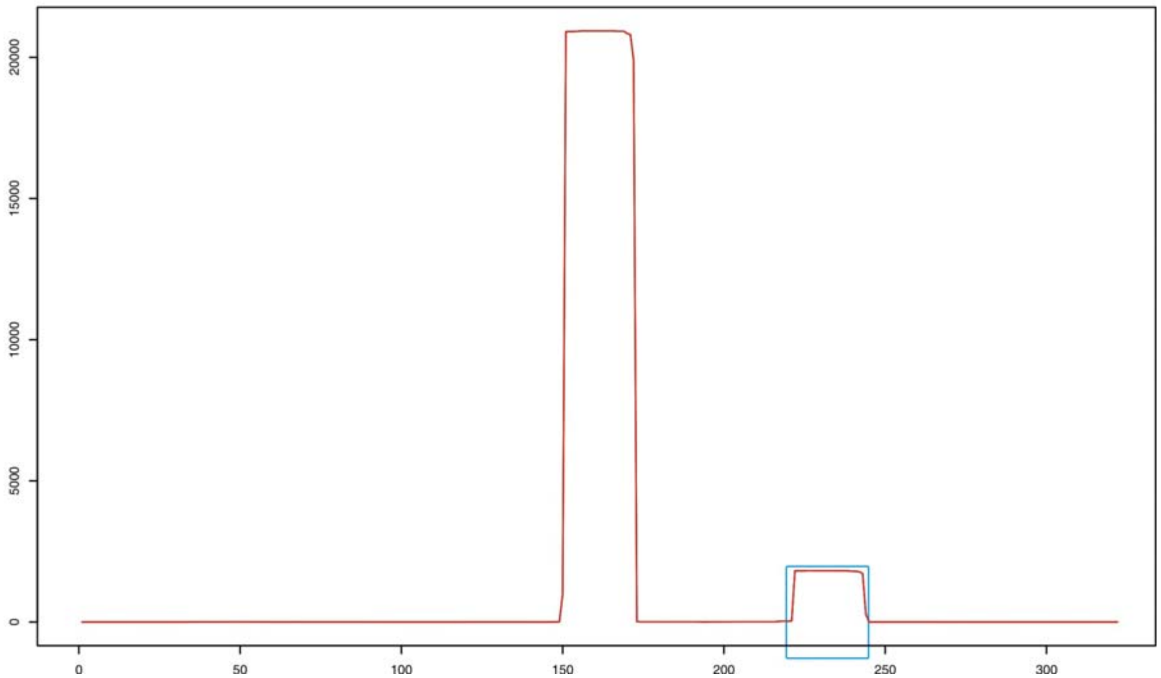

20000
15000
10000
5000
0
0
50
100
150
200
250
300

miRC5

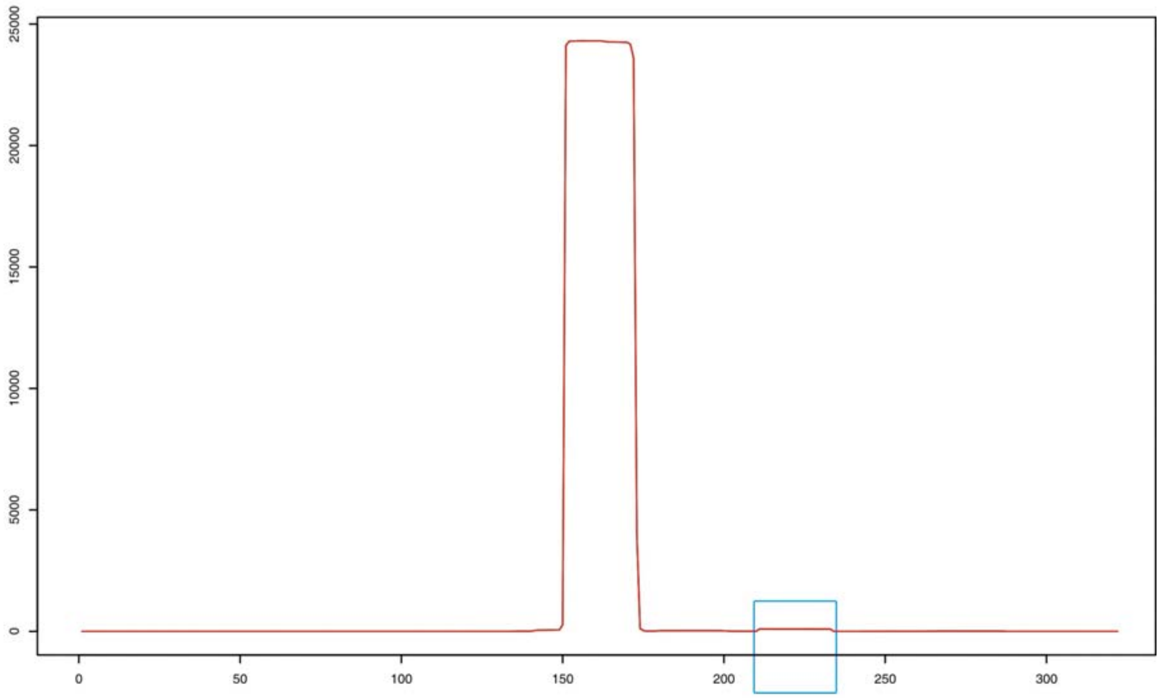

miRC6a

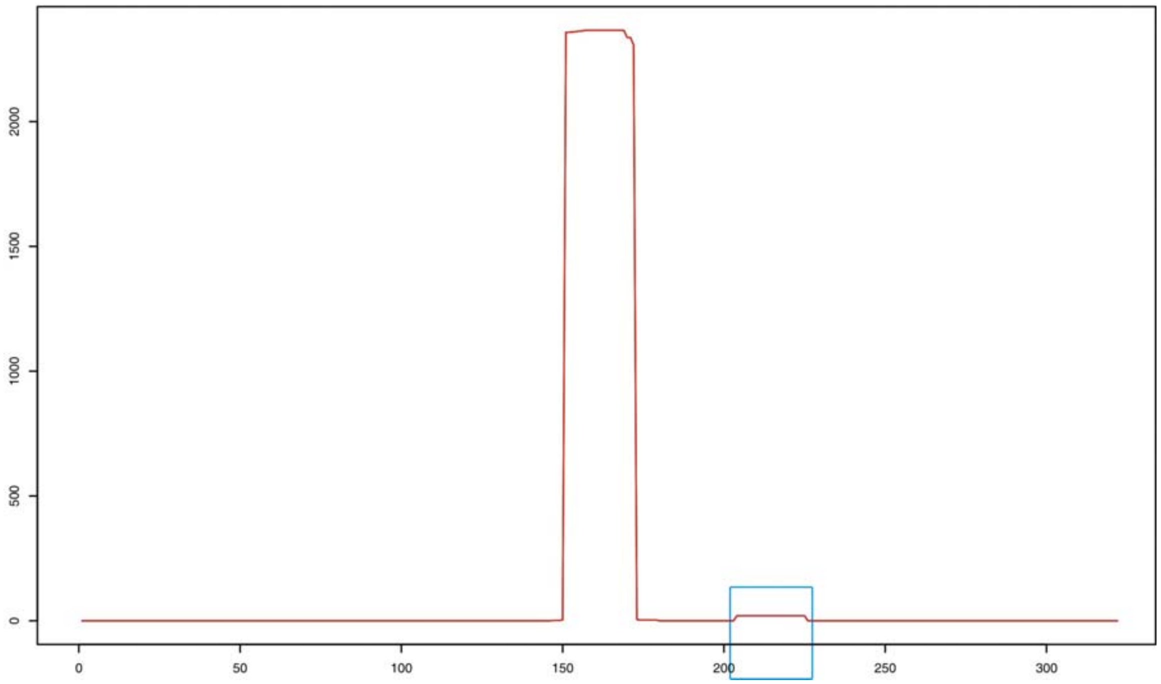

miRC6b

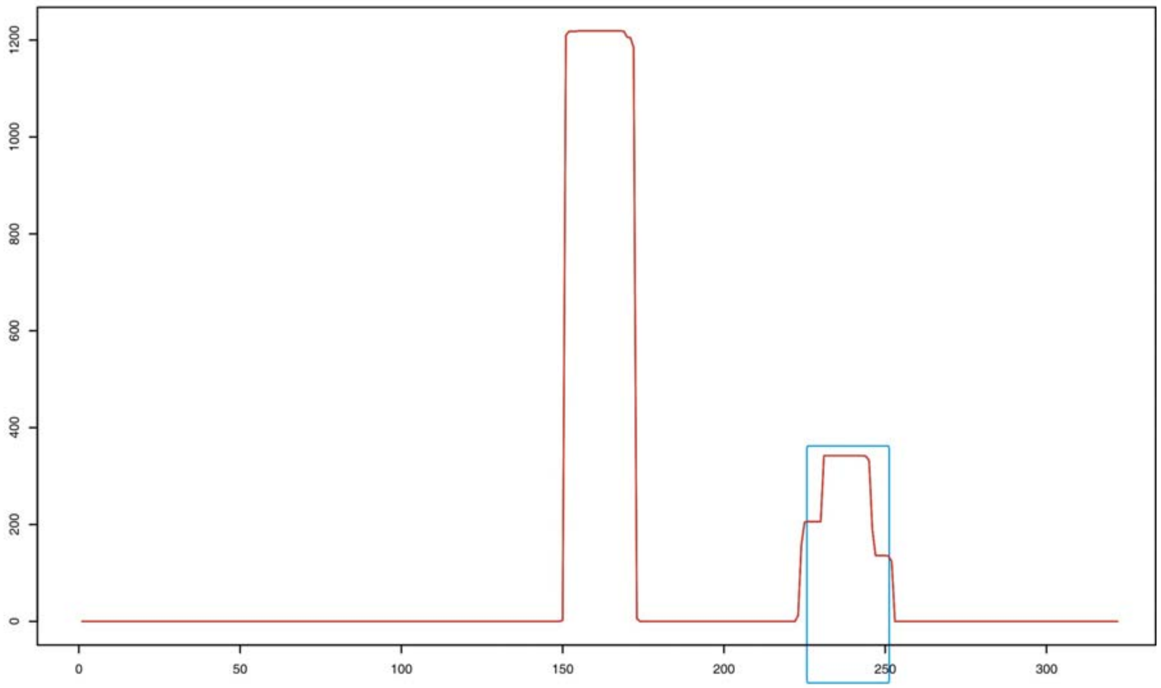

miRC7

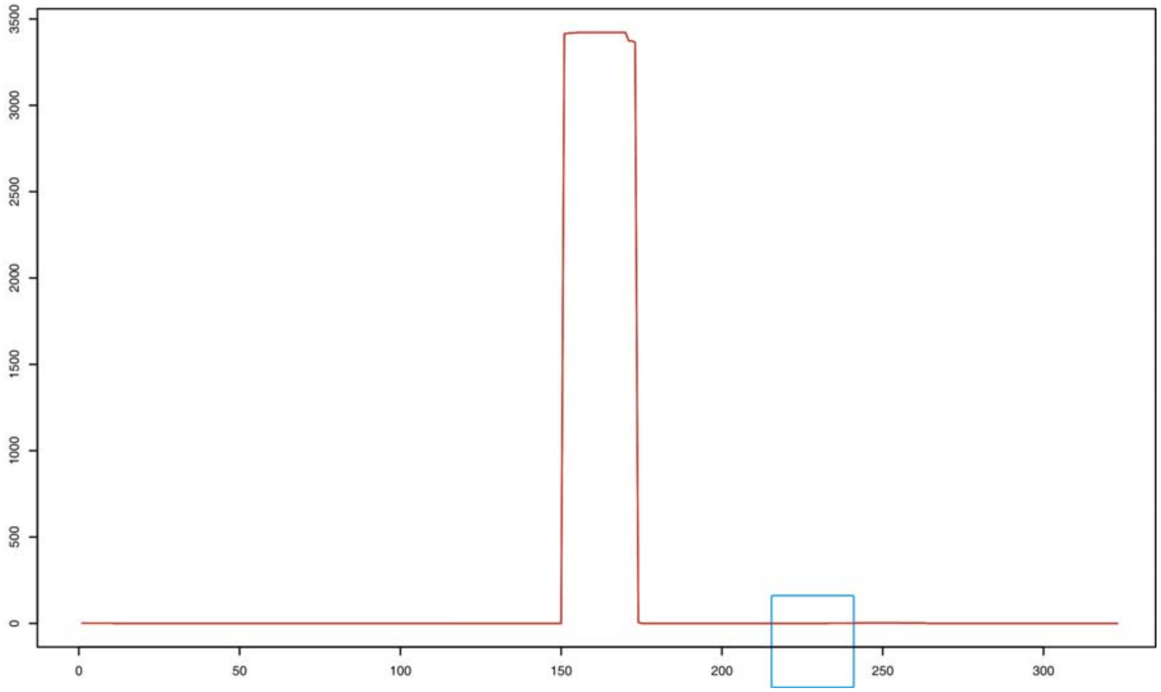

miRC8

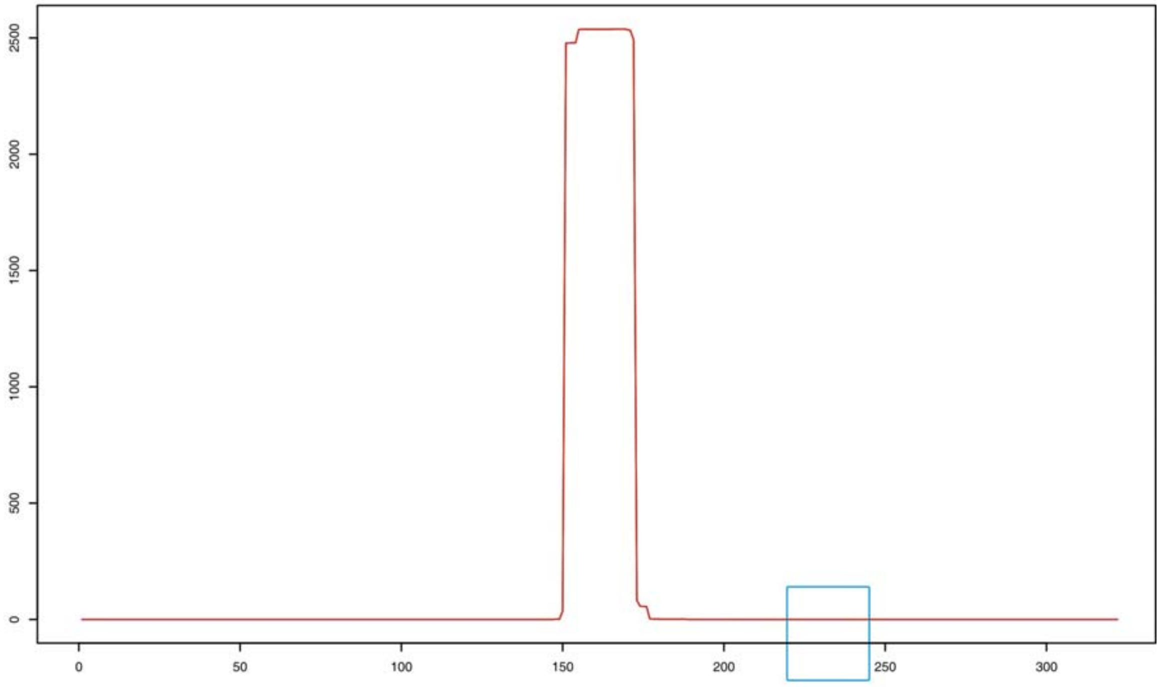

2500
2000
1500
1000
500
0
0
50
100
150
200
250
300

miRC9

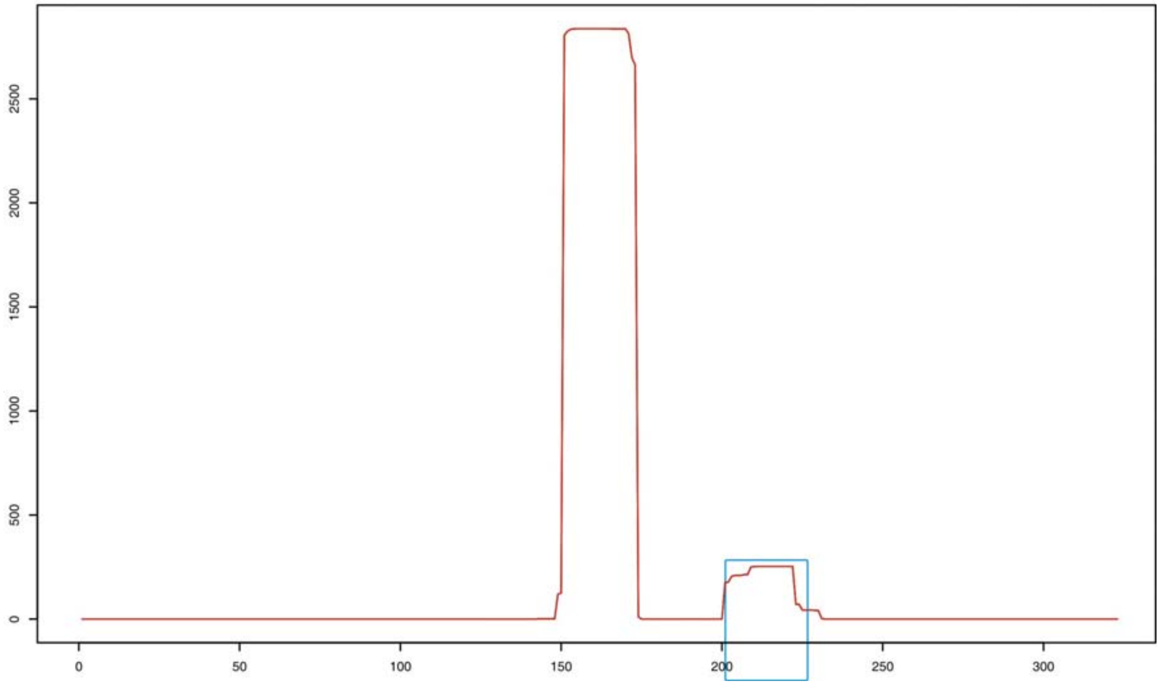

2500
2000
1500
1000
500
0
0
50
100
150
200
250
300

miRC10

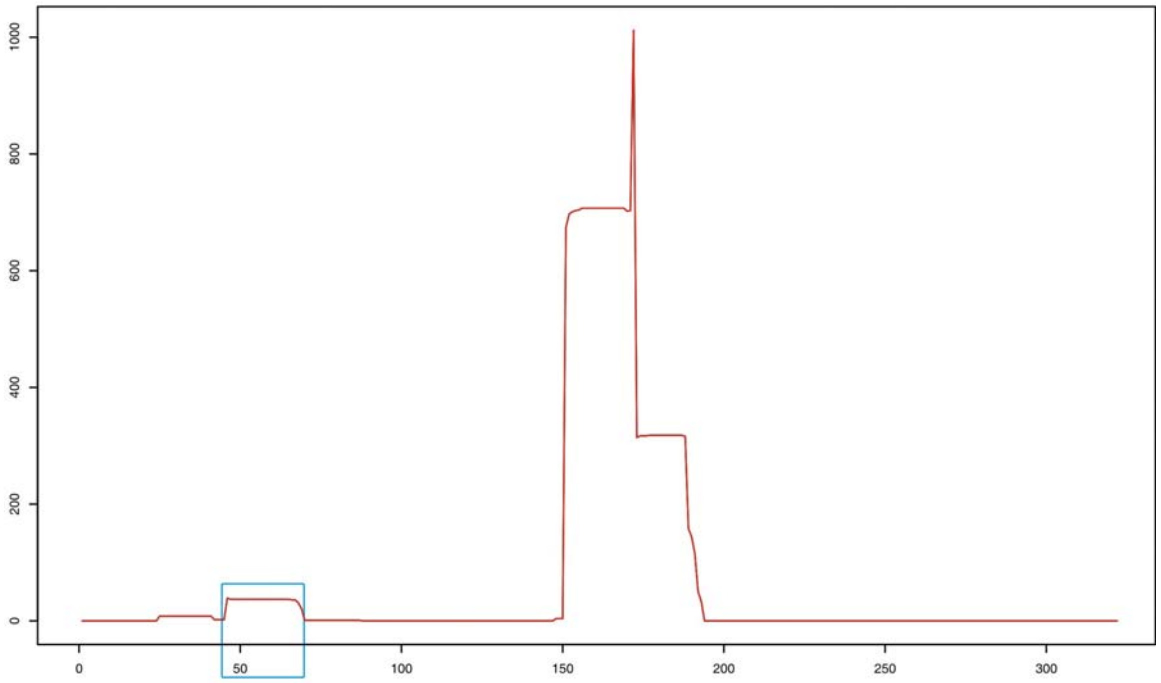

miRC11

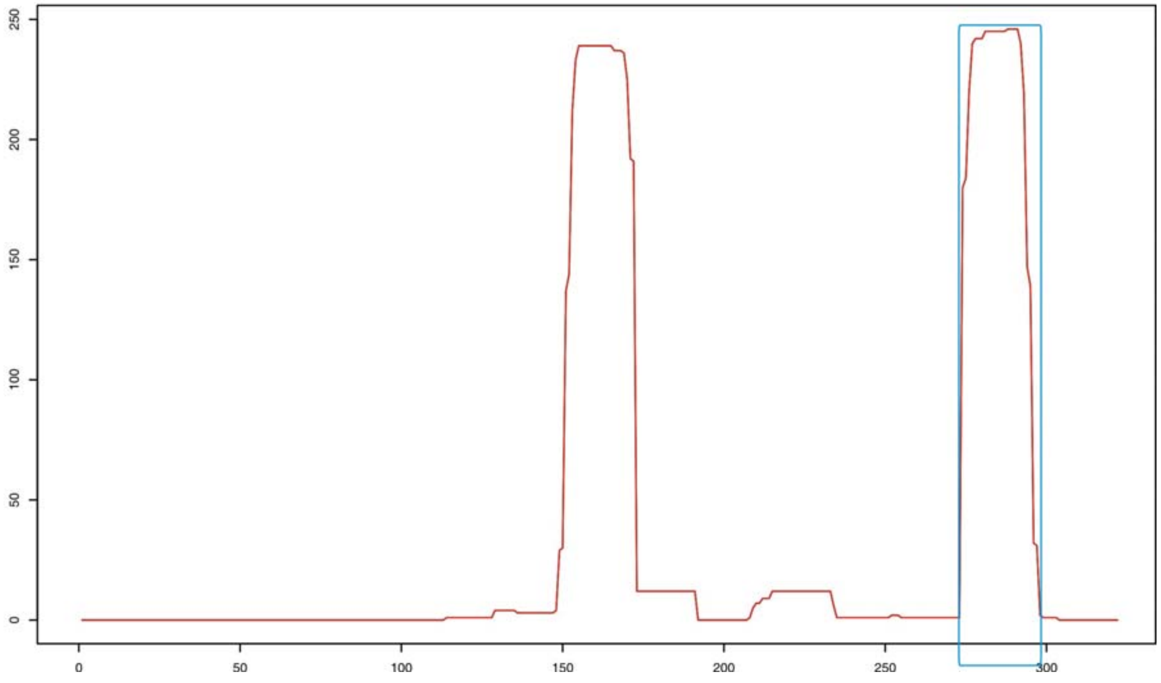

miRC12

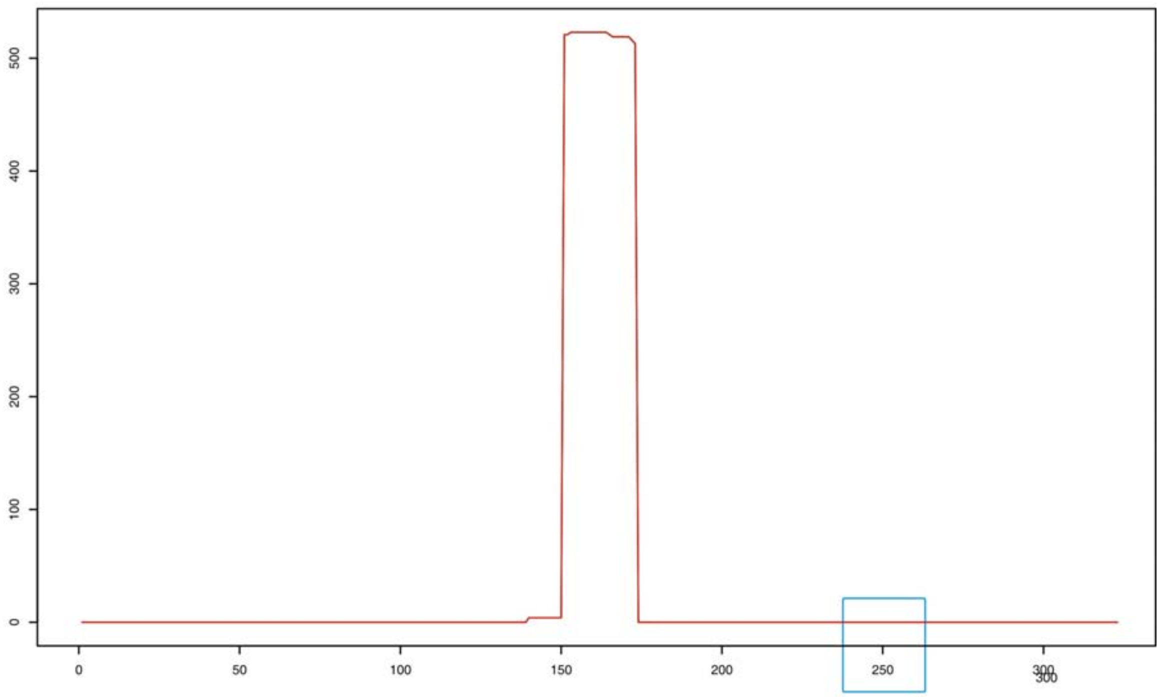

miRC13

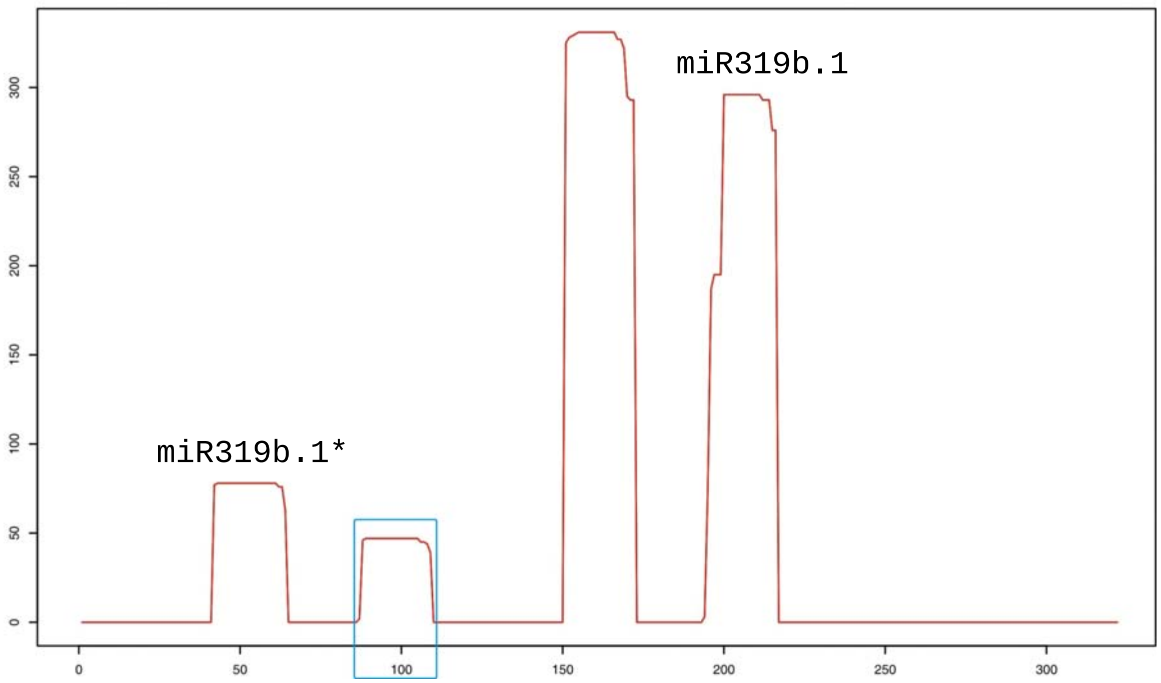

miRC14

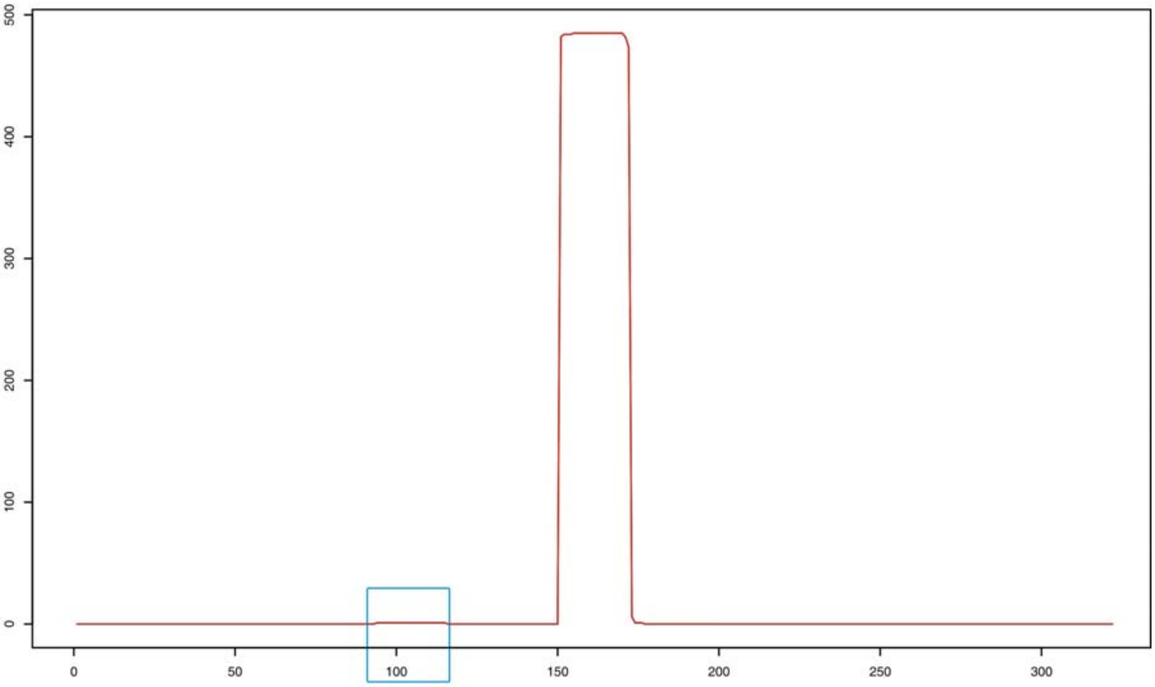

miRC15

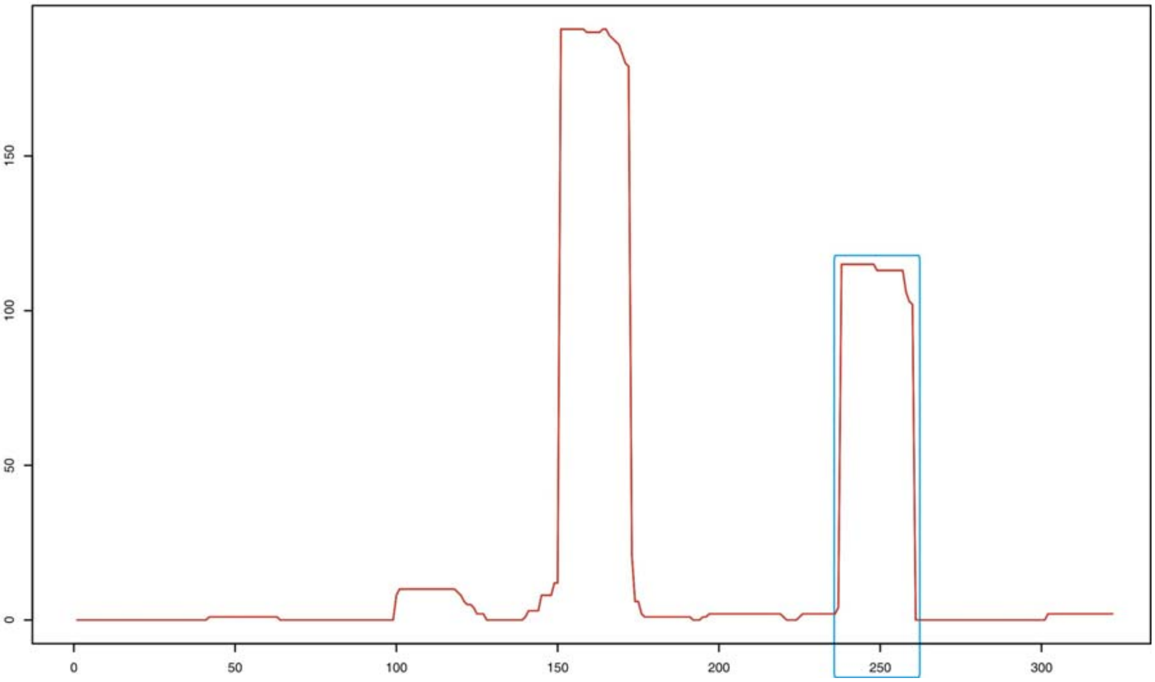

miRC16

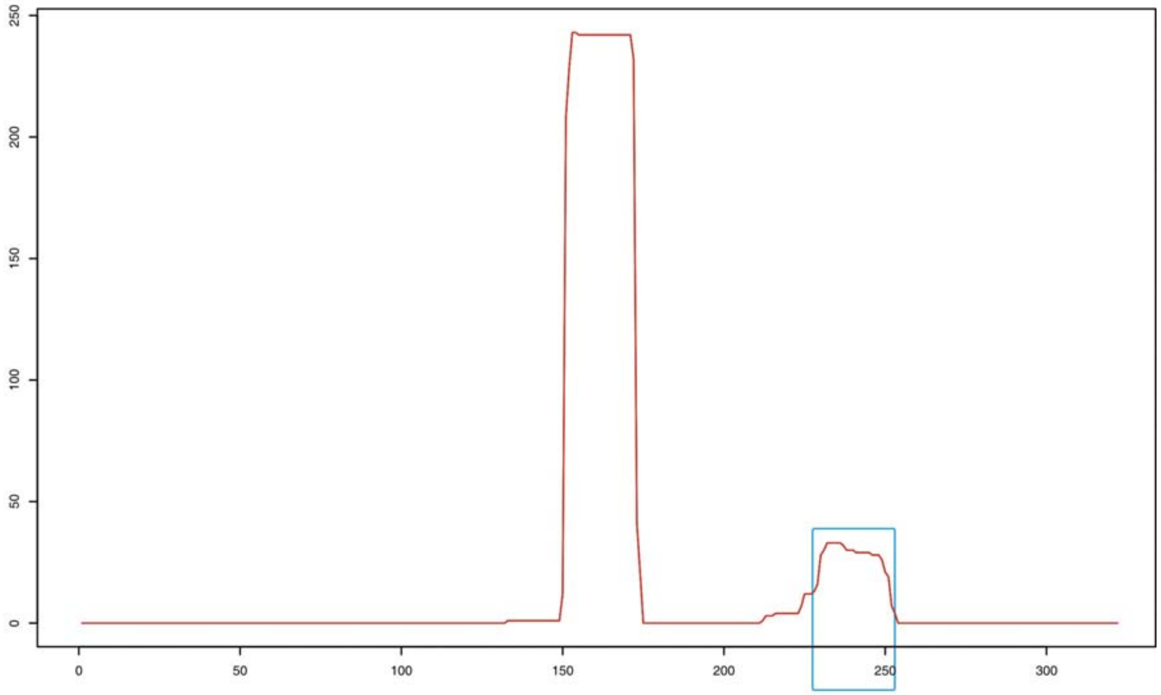

miRC17

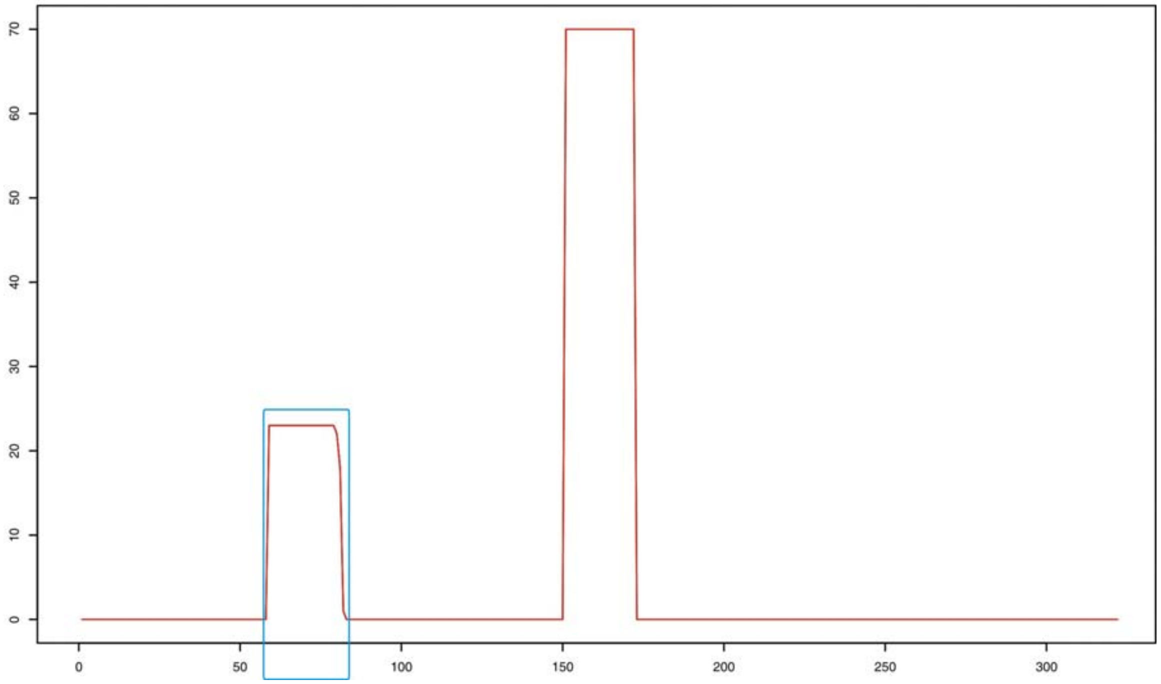

miRC18

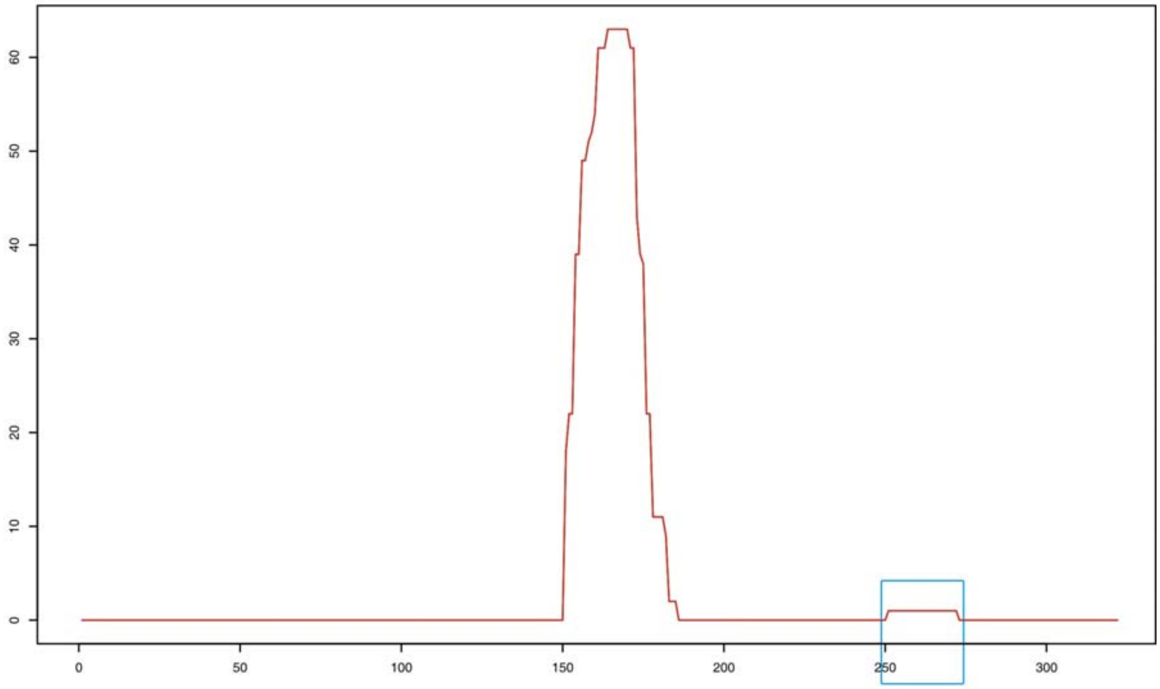

miRC19

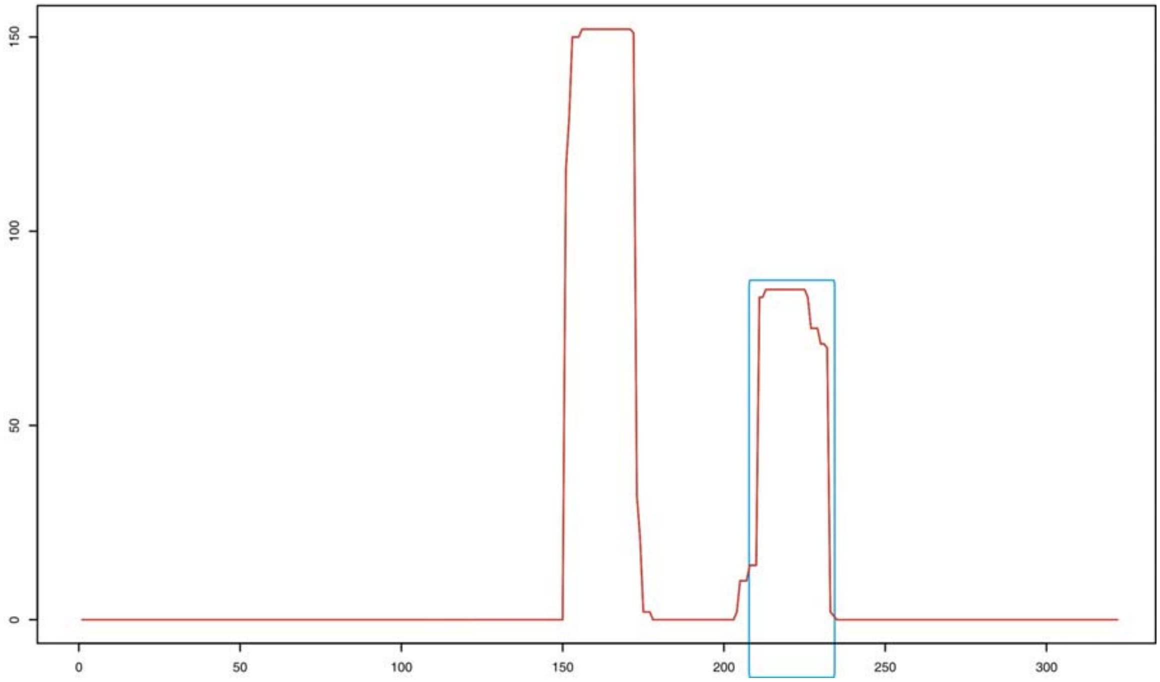

miRC20

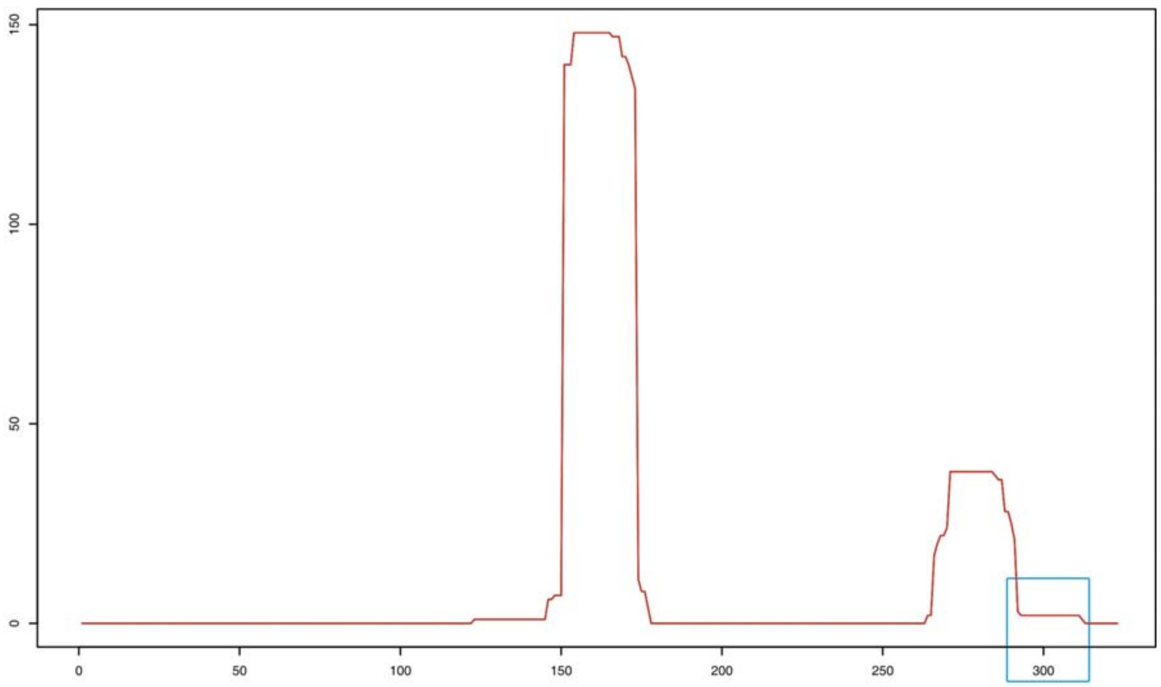

miRC21

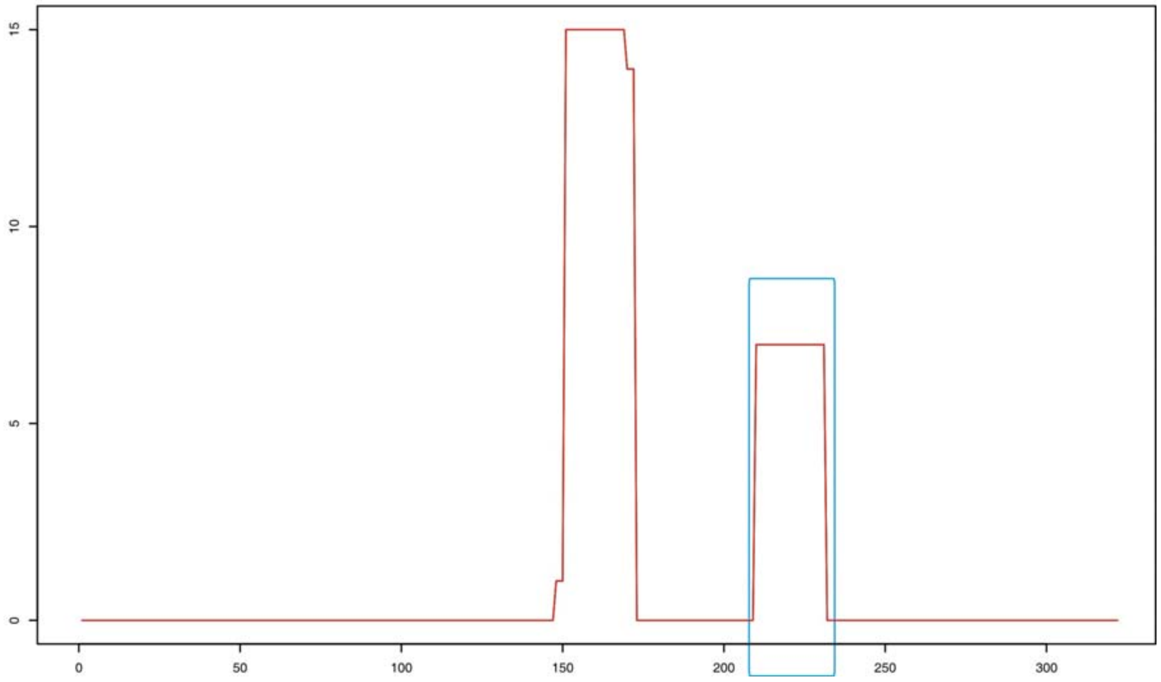

Supplement: Additional file 4 — Mapping plots of novel apple miRNAs. This file contains all the mapping plots illustrating the read distribution along the precursor region of novel apple miRNAs. [file gb-2012-13-6-r47-S4.PDF]
